# Supplementary material for: Bivalent intra-spike binding provides durability against emergent Omicron lineages: Results from a global consortium
Source: Cell Rep. 2023 Jan 12;42(1):112014. doi: 10.1016/j.celrep.2023.112014 (PMC9834171; doi:10.1016/j.celrep.2023.112014)
Supplement: Document S1. Figures S1–S4 [file mmc1.pdf]

Cell Reports, Volume *42*

Supplemental information

Bivalent intra-spike binding provides durability against emergent Omicron lineages: Results from a global consortium

Heather M. Callaway, Kathryn M. Hastie, Sharon L. Schendel, Haoyang Li, Xiaoying Yu, Jeremy Shek, Tierra Buck, Sean Hui, Dan Bedinger, Camille Troup, S. Moses Dennison, Kan Li, Michael D. Alpert, Charles C. Bailey, Sharon Benzeno, Jody L. Bonnevier, Jin-Qiu Chen, Charm Chen, Hyeseon Cho, Peter D. Crompton, Vincent Dussupt, Kevin C. Entzminger, Yassine Ezzyat, Jonathan K. Fleming, Nick Geukens, Amy E. Gilbert, Yongjun Guan, Xiaojian Han, Christopher J. Harvey, Julia M. Hatler, Bryan Howie, Chao Hu, Ailong Huang, Maya Imbrechts, Aishun Jin, Nik Kamachi, Gladys Keitany, Mark Klinger, Jay K. Kolls, Shelly J. Krebs, Tingting Li, Feiyan Luo, Toshiaki Maruyama, Michael A. Meehl, Letzibeth Mendez-Rivera, Andrea Musa, C.J. Okumura, Benjamin E.R. Rubin, Aaron K. Sato, Meiying Shen, Anirudh Singh, Shuyi Song, Joshua Tan, Jeffrey M. Trimarchi, Dhruvkumar P. Upadhyay, Yingming Wang, Lei Yu, Tom Z. Yuan, Erik Yusko, Bjoern Peters, Georgia Tomaras, and Erica Ollmann Saphire

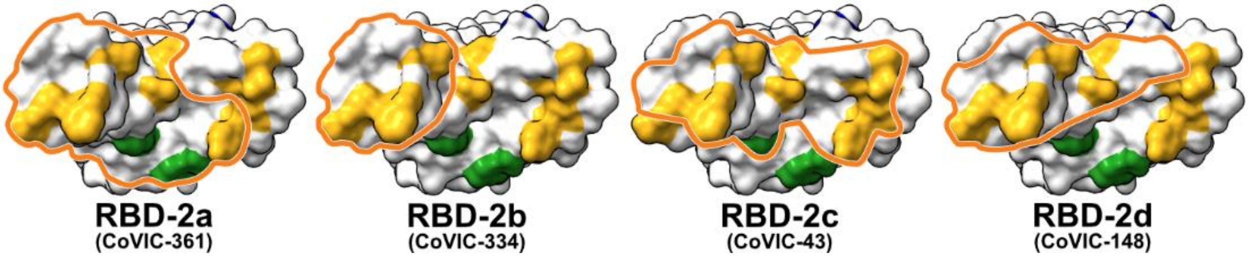

Figure S1. Antigenic sites of RBD-2 subgroups, related to Figure 1. Antigenic site footprints for RBD-2 subgroups RBD-2a, -2b, -2c, and -2d, approximated by docking a Fab into a negative stain electron microscopy map of spike bound to a representative antibody from the indicated epitope community, are highlighted by an orange outline.

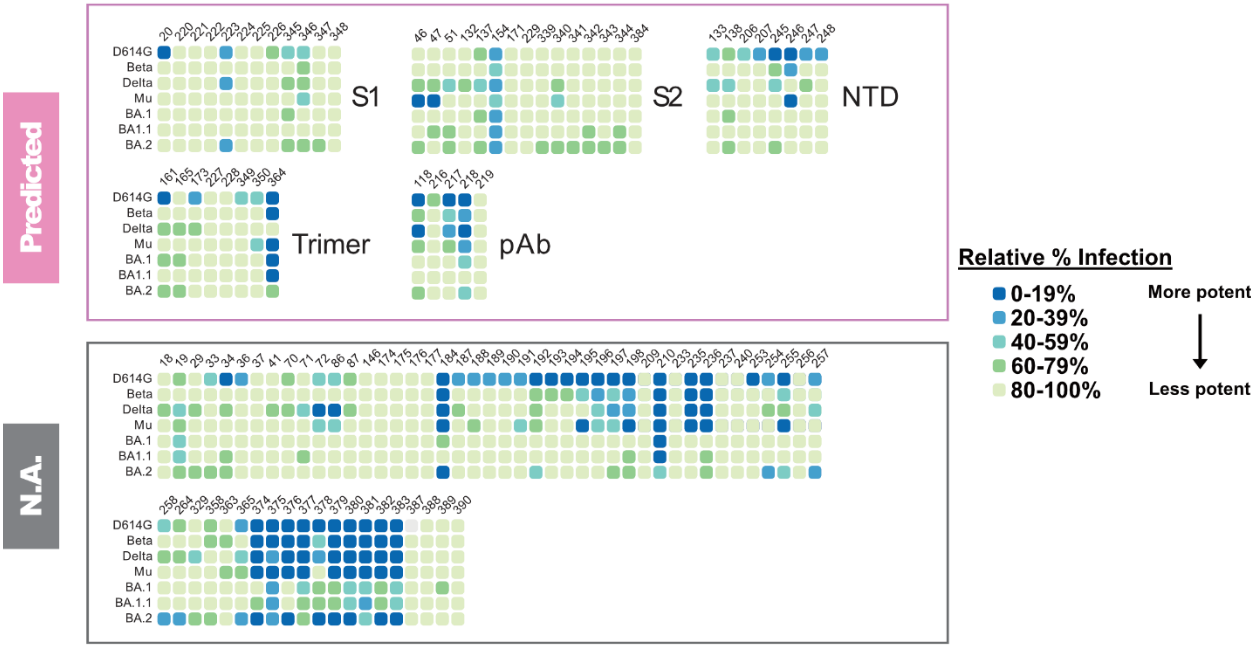

Figure S2. Block neutralization of SARS-CoV-2 pseudovirus bearing VoC spike proteins with additional CoVIC panel antibodies, related to Figure 2. Neutralization of SARS-CoV-2 pseudovirus with 250ng/mL antibody. Antibodies are organized by their predicted binding site or are uncategorized. Neutralization activity is expressed as the percentage of infected cells in the presence of antibody relative to cells infected in the absence of antibody.

A

RBD		IC50 (ng/mL) D614G	BA2.12.1	BA.4/5
1	COVIC-89	316.1	121.8	100.2
1	COVIC-93	26.6	30.8	39.0
1	COVIC-234	131.4	62.8	55.6
2a	COVIC-294	8.3	16.8	2.3
2a	COVIC-299	47.8	31.7	21.8
2a	COVIC-359	7.5	31.2	>>2,000
2a	COVIC-360	13.0	36.3	>>2,000
2a	COVIC-361	28.7	32.0	>2,000*
2a	COVIC-362	6.6	9.9	>2,000*
2b	COVIC-102	4.8	15.3	>2,000*
2b	COVIC-103	3.0	10.4	>2,000*
2b	COVIC-290	4.6	10.6	>2,000*
2b	COVIC-292	1.8	38.0	>>2,000
2b	COVIC-333	4.7	9.6	>>2,000
2b	COVIC-334	7.1	34.4	>2,000*
2b	COVIC-354	16.6	104.0	>>2,000
3a	COVIC-368	2.5	10.8	5.4
4a	COVIC-371	1.0	11.2	>2,000*
4b	COVIC-295	25.2	44.3	>2,000*
5d	COVIC-355	0.9		11.5

*Some neutralization observed at higher antibody concentrations

B

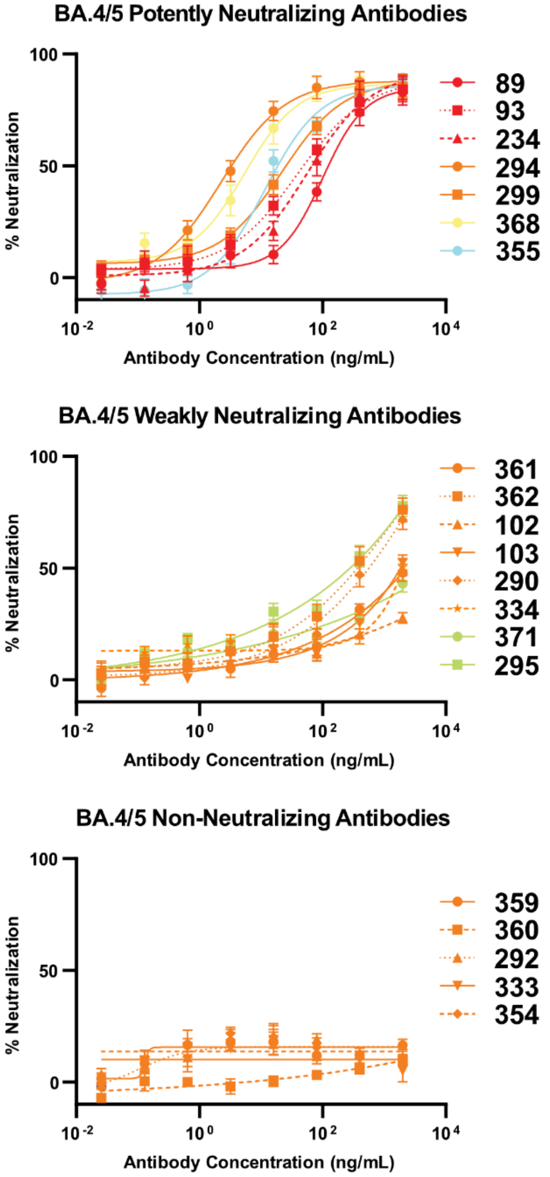

Figure S3. Omicron BA.4/5 and BA2.12.1 pseudovirus neutralization by select antibodies that potently neutralize Omicron BA.1, BA1.1, and BA.2, related to Figures 2 and 4. A) Neutralization titers against pseudovirus bearing D614G, BA2.12.1, and BA.4/5 spike for CoVIC antibodies that potently neutralized Omicron BA.1, BA1.1, and BA.2 in the block neutralization assay. **B)** Neutralization curves for BA.4/5 pseudoviruses. Error bars are ± SEM.

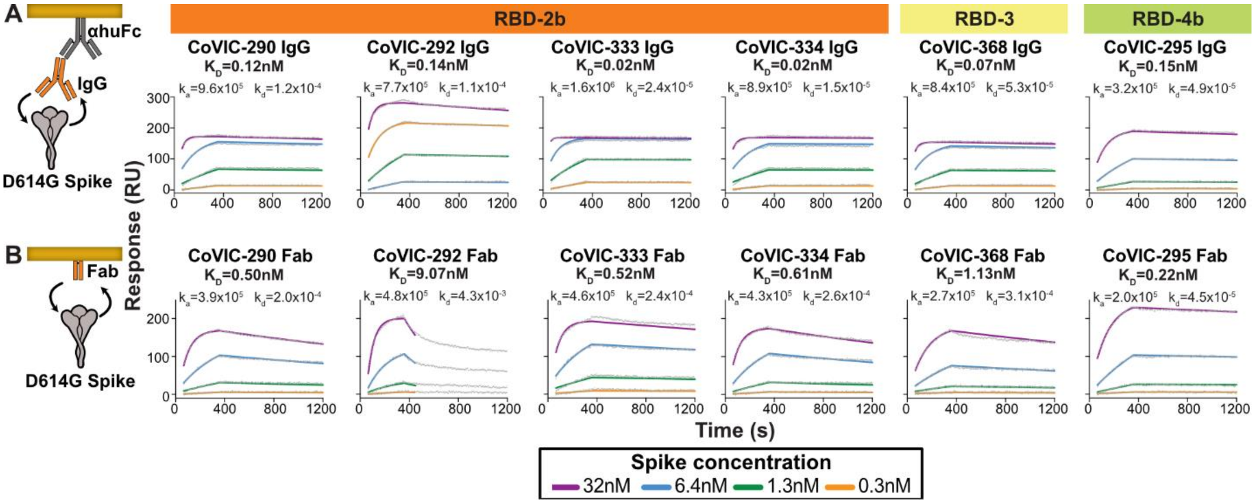

Figure S4. Binding kinetics of D614G spike ectodomains to IgG and Fabs, related to Figure 5. Surface plasmon resonance experiments comparing the binding kinetics of IgG **(A)** and Fab **(B)** to D614G spike. For each sample, experimental data (gray line) and 1:1 fitted curve (colored lines) are shown. Spike concentrations range from 0.3-32nM. K_D, k_a, and k_d values for each interaction are indicated.

Table S1. Neutralization activity of all items in the CoVIC panel against pseudovirus bearing major SARS-CoV-2 VoCs, related to Figures 2 and S2. Neutralization activity is expressed as the percentage of infected cells in the presence of 250ng/mL or 25μg/mL antibody relative to cells infected in the absence of antibody.
