# Supplementary material for: Bivalent intra-spike binding provides durability against emergent Omicron lineages: Results from a global consortium
Source: Cell Rep. 2023 Jan 12;42(1):112014. doi: 10.1016/j.celrep.2023.112014 (PMC9834171; doi:10.1016/j.celrep.2023.112014)
Supplement: Document S2. Article plus supplemental information [file mmc3.pdf]

Cell Reports

Article

Bivalent intra-spike binding provides durability against emergent Omicron lineages: Results from a global consortium

Graphical abstract

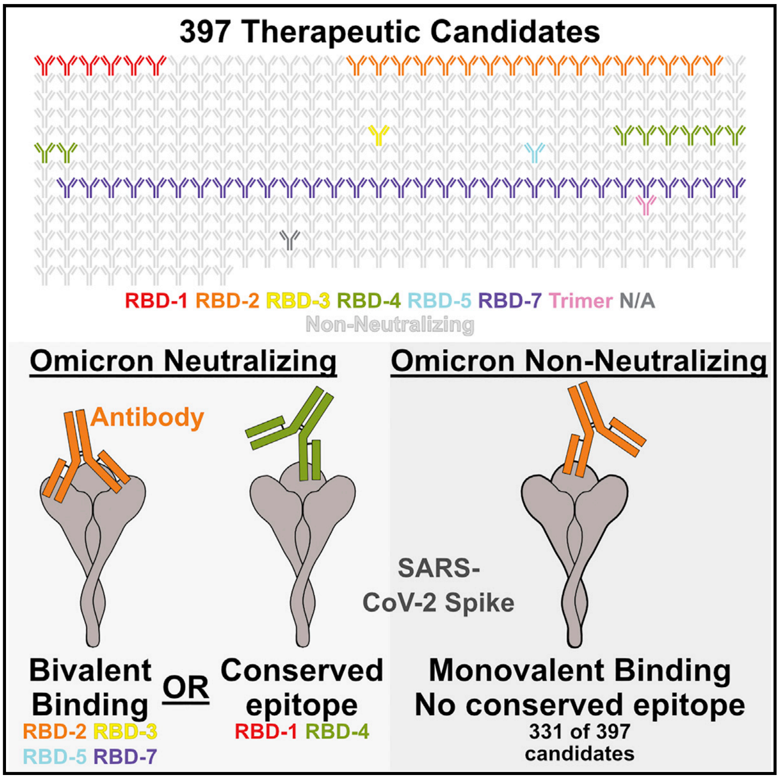

Highlights

- 66/397 of CoVIC IgGs raised against earlier SARS-CoV-2 also neutralize Omicron
- 14 of these recognize epitopes conserved in Omicron
- Most others recognize highly mutated epitopes, retaining activity by binding bivalently
- Cleavage of bivalent binding IgGs to Fabs abolishes Omicron neutralization

Authors

Heather M. Callaway, Kathryn M. Hastie, Sharon L. Schendel, ..., Bjoern Peters, Georgia Tomaras, Erica Ollmann Saphire

Correspondence

erica@lji.org

In brief

Callaway et al. examine a panel of 397 SARS-CoV-2 antibodies to understand how some antibodies, generated early during the COVID-19 pandemic, maintain the ability to neutralize Omicron variants despite extensive mutation in their epitopes. Antibodies against highly mutated epitopes retain neutralization by binding bivalently: cleavage to Fab eliminates neutralization.

Callaway et al., 2023, Cell Reports *42*, 112014
January 31, 2023 © 2023 The Author(s).
https://doi.org/10.1016/j.celrep.2023.112014

CellPress

Cell Reports

CellPress OPEN ACCESS

Article

Bivalent intra-spike binding provides durability against emergent Omicron lineages: Results from a global consortium

Heather M. Callaway,[1] Kathryn M. Hastie,[1] Sharon L. Schendel,[1] Haoyang Li,[1] Xiaoying Yu,[1] Jeremy Shek,[1] Tierra Buck,[1] Sean Hui,[1] Dan Bedinger,[2] Camille Troup,[2] S. Moses Dennison,[3] Kan Li,[3] Michael D. Alpert,[4] Charles C. Bailey,[4] Sharon Benzeno,[5] Jody L. Bonnevier,[6] Jin-Qiu Chen,[7] Charm Chen,[7] Hyeseon Cho,[8,9] Peter D. Crompton,[8,9] Vincent Dussupt,[10,11] Kevin C. Entzminger,[12] Yassine Ezzyat,[13] Jonathan K. Fleming,[12] Nick Geukens,[14] Amy E. Gilbert,[5] Yongjun Guan,[15,16] Xiaojian Han,[17] Christopher J. Harvey,[13,18] Julia M. Hatler,[6] Bryan Howie,[5] Chao Hu,[17] Ailong Huang,[19]

(Author list continued on next page)

[1]Center for Infectious Disease and Vaccine Research, La Jolla Institute for Immunology, La Jolla, CA 92037, USA
[2]Carterra, 825 N. 300 W. Ste. C309, Salt Lake City, UT 84103, USA
[3]Center for Human Systems Immunology, Departments of Surgery, Immunology, and Molecular Genetics and Microbiology and Duke Human Vaccine Institute, Duke University, Durham, NC 27701, USA
[4]Emmune, Inc., 14155 US Highway 1, Juno Beach, FL 33408, USA
[5]Adaptive Biotechnologies, 1551 Eastlake Ave East, Seattle, WA 98102, USA
[6]Bio-techne, 614 McKinley Place NE, Minneapolis, MN 55413, USA
[7]ACRO Biosystems, 1 Innovation Way, Newark, DE 19711, USA
[8]Antibody Biology Unit, Laboratory of Immunogenetics, National Institute of Allergy and Infectious Diseases, National Institutes of Health, Rockville, MD 20852, USA
[9]Malaria Infection Biology and Immunity Section, Laboratory of Immunogenetics, National Institute of Allergy and Infectious Diseases, National Institutes of Health, Rockville, MD 20852, USA
[10]Emerging Infectious Diseases Branch, Walter Reed Army Institute of Research, Silver Spring, MD, USA
[11]Henry M. Jackson Foundation for the Advancement of Military Medicine, Bethesda, MD, USA
[12]Abwiz Bio, Inc., 9823 Pacific Heights Blvd. Suite J, San Diego, CA 92121, USA
[13]Jounce Therapeutics, Inc., 780 Memorial Drive, Cambridge, MA 02139, USA
[14]PharmAbs, The KU Leuven Antibody Center, KU Leuven, 3000 Leuven, Belgium
[15]Antibody BioPharm, Inc., 401 Professional Dr Ste 241, Gaithersburg, MD 20879, USA

(Affiliations continued on next page)

SUMMARY

The SARS-CoV-2 Omicron variant of concern (VoC) and its sublineages contain 31–36 mutations in spike and escape neutralization by most therapeutic antibodies. In a pseudovirus neutralization assay, 66 of the nearly 400 candidate therapeutics in the Coronavirus Immunotherapeutic Consortium (CoVIC) panel neutralize Omicron and multiple Omicron sublineages. Among natural immunoglobulin Gs (IgGs), especially those in the receptor-binding domain (RBD)-2 epitope community, nearly all Omicron neutralizers recognize spike bivalently, with both antigen-binding fragments (Fabs) simultaneously engaging adjacent RBDs on the same spike. Most IgGs that do not neutralize Omicron bind either entirely monovalently or have some (22%–50%) monovalent occupancy. Cleavage of bivalent-binding IgGs to Fabs abolishes neutralization and binding affinity, with disproportionate loss of activity against Omicron pseudovirus and spike. These results suggest that VoC-resistant antibodies overcome mutagenic substitution via avidity. Hence, vaccine strategies targeting future SARS-CoV-2 variants should consider epitope display with spacing and organization identical to trimeric spike.

INTRODUCTION

Since emerging in late 2019, SARS-CoV-2, the causative agent of COVID-19, has infected over 550 million people worldwide.[1] In the United States alone, over 1 million people have died from COVID-19, and over 6 million people have died worldwide.[1,2] As of July 2022, 10 vaccines received WHO emergency use authorization (EUA), and more than 12 billion doses have been delivered.[3] However, a large proportion of the world's population remains unvaccinated. Current vaccines are substantially

This is an open access article under the CC BY license (http://creativecommons.org/licenses/by/4.0/).

Maya Imbrechts,[14] Aishun Jin,[17] Nik Kamachi,[7] Gladys Keitany,[5] Mark Klinger,[5] Jay K. Kolls,[20] Shelly J. Krebs,[10,11] Tingting Li,[17] Feiyan Luo,[17] Toshiaki Maruyama,[12] Michael A. Meehl,[13] Letzibeth Mendez-Rivera,[10,11] Andrea Musa,[5] C.J. Okumura,[12] Benjamin E.R. Rubin,[5] Aaron K. Sato,[21] Meiying Shen,[22] Anirudh Singh,[13] Shuyi Song,[17] Joshua Tan,[8] Jeffrey M. Trimarchi,[4,23] Dhruvkumar P. Upadhyay,[13,24] Yingming Wang,[17] Lei Yu,[25] Tom Z. Yuan,[21] Erik Yusko,[5] Bjoern Peters,[1,26] Georgia Tomaras,[3] and Erica Ollmann Saphire[1,26,27,*]

[16]Shanghai Life Technology Co., Ltd., 781 Cai Lun Rd, Ste 801, Pudong, Shanghai 201203, China
[17]Department of Immunology, College of Basic Medicine, Chongqing Medical University, Chongqing 400010, China
[18]Phenomic AI, 661 University Avenue, Suite 1300 MaRS Centre, West Tower, Toronto, ON M5G 0B7, Canada
[19]Key Laboratory of Molecular Biology for Infectious Diseases (Ministry of Education), Institute for Viral Hepatitis, Department of Infectious Diseases, The Second Affiliated Hospital, Chongqing Medical University, Chongqing 400010, China
[20]Tulane School of Medicine, Center for Translational Research in Infection and Inflammation, New Orleans, LA 70112, USA
[21]Twist Bioscience, 681 Gateway Blvd., South San Francisco, CA 94080, USA
[22]Department of Endocrine Breast Surgery, The First Affiliated Hospital of Chongqing Medical University, Chongqing 400010, China
[23]Department of Biological Sciences, Lehigh University, 111 Research Drive, Bethlehem, PA 18015, USA
[24]Amgen, Inc., 360 Binney St., Cambridge, MA 02141, USA
[25]Guangzhou Eighth People's Hospital & Guangzhou Medical University, Guangzhou 510060, China
[26]Department of Medicine, University of California San Diego, La Jolla, CA 92039, USA
[27]Lead contact
*Correspondence: erica@lji.org
https://doi.org/10.1016/j.celrep.2023.112014

less effective against Omicron and emerging SARS-CoV-2 variants, although bivalent formulations that recently received EUA may provide better protection.[4] Vaccines still reduce disease severity, but with continued high infection rates, individuals who are immunocompromised, who cannot be vaccinated, or who have other risk factors associated with severe disease will need access to prophylactic and therapeutic interventions that retain efficacy. Monoclonal antibody (mAb) therapeutics against the SARS-CoV-2 spike protein are a key prophylactic or therapeutic option for at-risk patients and for vaccine breakthrough. During the course of infection, antibodies mechanically neutralize virions by blocking receptor binding, crosslinking viral proteins, preventing fusion with the host cell membrane, or targeting virions and infected cells for immune clearance. However, the multiple mutations in the SARS-CoV-2 spike protein of the Omicron variant have rendered most existing mAb therapeutics ineffective.

Omicron BA.1 (also known as B.1.1.529) was first reported to the World Health Organization in late November 2021 and was declared a variant of concern (VoC) days later.[5,6] Omicron's high transmission rate ($R_0 > 3$) and rapid doubling time contributed to its becoming the dominant VoC within about 5 weeks of its discovery.[2,3] With 62 non-synonymous mutations (36 in spike), Omicron has substantially more mutations than Alpha (24 mutations, 10 in spike), Beta (20 and 10), Gamma (24 and 10), or Delta VoCs (22 and 9) (Figure 1). Of the 36 mutations in the Omicron spike protein, 10 are in the N-terminal domain (NTD), and 11 are in the S2 subunit, which controls fusion. The remaining 15 mutations are all in the receptor-binding domain (RBD), which interacts with the angiotensin-converting enzyme 2 (ACE2) receptor. These RBD mutations include G339D, S371L, S373P, S375F, K417N, N440K, G446S, S477N, T478K, E484A, Q493R, G496S, Q498R, N501Y, and Y505H (Figure 1). Despite these substitutions, the Omicron spike still binds ACE2 with high affinity.[7,8]

The BA.1.1 sublineage emerged largely in parallel with BA.1, with an array of essentially identical mutations, but carries an R346K mutation and lacks the K417N mutation in the RBD. BA.1 and BA1.1 co-dominated until mid-February 2022, when another sublineage, BA.2, began to surge. BA.2 has several new mutations in both the RBD and NTD (including T367A, D405N, and R408S in the RBD) but lacks the G446S and G496S mutations of BA.1. and BA.1.1. The sublineages BA.4 and BA.5, which contain L452R and F486V mutations in the RBD but lack Q493R and G496S, are now the dominant source of new infections for which virus sequence information is available.[9] BA.2.12.1, another Omicron lineage, has a spike sequence similar to that of BA.4 and BA.5. but has Q493R and L452Q RBD mutations and lacks the F486V mutation.

In addition to rendering most existing antibody therapeutics ineffective, Omicron spike mutations also challenged host antibody responses. Sera from individuals who received two doses of mRNA-based vaccines were 25-fold less potent against Omicron,[10–13] and most vaccinated individuals remain susceptible to Omicron infection.[14] Because SARS-CoV-2 likely will continue to accumulate mutations as it adapts to human hosts and undergoes selective pressure, vaccines and antibody therapies that are mutation resistant are urgently needed. Achieving durability requires better understanding of how some antibodies maintain neutralization capacity in the face of numerous point mutations in their epitopes.

To identify Omicron-neutralizing antibodies, we tested 397 samples contributed to the Coronavirus Immunotherapeutic Consortium (CoVIC) panel for neutralization against pseudoviruses displaying spike from Omicron and its sublineages. The CoVIC is an international effort designed to broadly characterize the antibody landscape against SARS-CoV-2 and compare candidate antibody therapeutics side by side.[15] The CoVIC panel comprises contributions from 60 different companies or academic laboratories across four continents. Included in the panel are natural monoclonal immunoglobulin Gs (IgGs), bispecific antibodies, nanobodies, polyclonal products, and engineered multivalent binding constructs. Multiple approaches were used to generate the candidate therapeutics in the panel, which originated from survivors of COVID-19, survivors of SARS-CoV-1 in 2003, or immunized wild-type and humanized mice or were engineered using *in silico* methods.

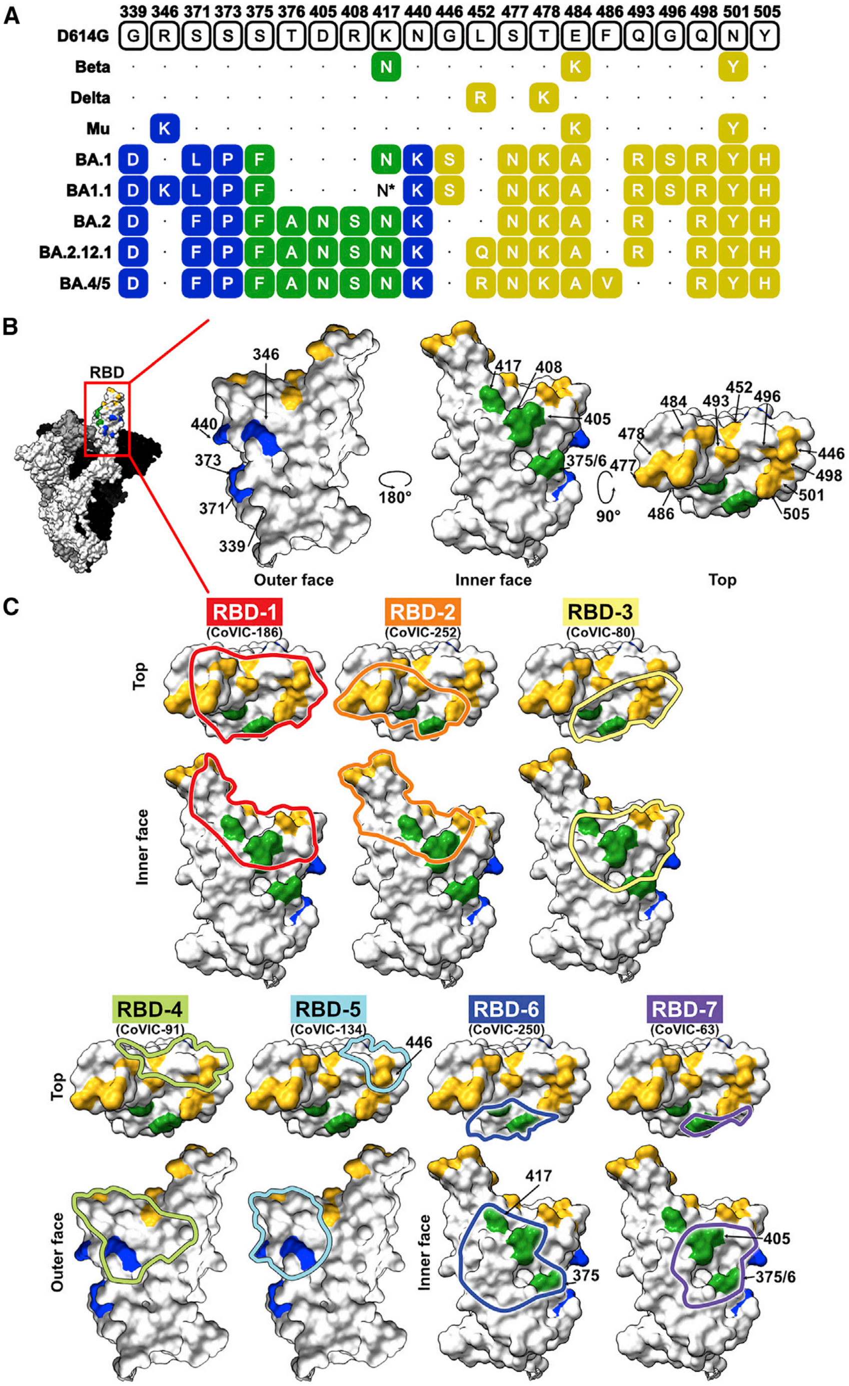

Figure 1. RBD point mutations in SARS-CoV-2 VoC spike proteins and antigenic sites

(A) Sequence alignment showing point mutations in the RBD of the indicated VoC.

(B) Molecular models of the RBD with point mutations on the outer face (blue), inner face (green), and top (yellow) highlighted (PDB: 7A94).

(C) Binding footprints are outlined in colors corresponding to the RBD antigenic site and were approximated by docking a Fab into a negative-stain electron microscopy map of spike bound to a representative antibody from the indicated epitope community.

*The BA1.1 clones used in this study contain the K417N mutation in the RBD, but the consensus sequence for all BA1.1 isolates does not.

See also Figure S1.

Antibodies in the CoVIC were analyzed in a high-throughput, high-resolution competition analysis using surface plasmon resonance to sort the panel into competition groups, particularly those antibodies that target the RBD. These analyses identified seven major epitope communities among RBD-reactive antibodies, termed RBD-1–7 (Figures 1 and S1). The seven communities and their subgroups provide finer divisions than previous characterizations and identify important functional differences.

RBD-1 overlaps the receptor-binding motif (RBM) almost exactly, whereas RBD-2 antibodies target an epitope that is shifted slightly toward the outer face relative to RBD-1. Antibodies in the RBD-3 community have footprints shifted toward N501. Together, RBD-1–3 correspond to functional subdivisions of the "class 1" antibodies described by Barnes et al.[16] RBD-4 and -5 antibodies both bind the outer face of RBD and are similar to class 2 and class 3, respectively. Meanwhile, RBD-6 and -7 are related to class 4 but are functional subdivisions of that class, according to their competition profile.

We previously described RBD epitope communities for 176 CoVIC antibodies[15] and have since assigned 195 more antibodies to epitope communities, which resulted in a more finely detailed classification scheme. Now, all epitope communities except for RBD-1 and -3 have several subclusters that were defined based on their competition with other antibody communities. In particular, RBD-4 and -5 epitope communities now have additional subdivisions, and RBD-2b.1, -2b.2, and -2b.3 communities have been renamed as RBD-2c, -2d, and -2b, respectively, to better highlight their distinct epitope subgroups and relationship to other communities.

To determine what types of antibodies in the therapeutic landscape retain neutralization activity against Omicron subvariants and understand how epitope community is related to potency against Omicron, we tested the CoVIC antibodies in a neutralization assay using pseudovirus bearing Omicron spikes from BA.1, BA.2, and the sublineage BA1.1. A subset of antibodies that potently neutralized all three pseudoviruses were further tested against BA.4/BA.5 and sublineage BA2.12.1 pseudoviruses to

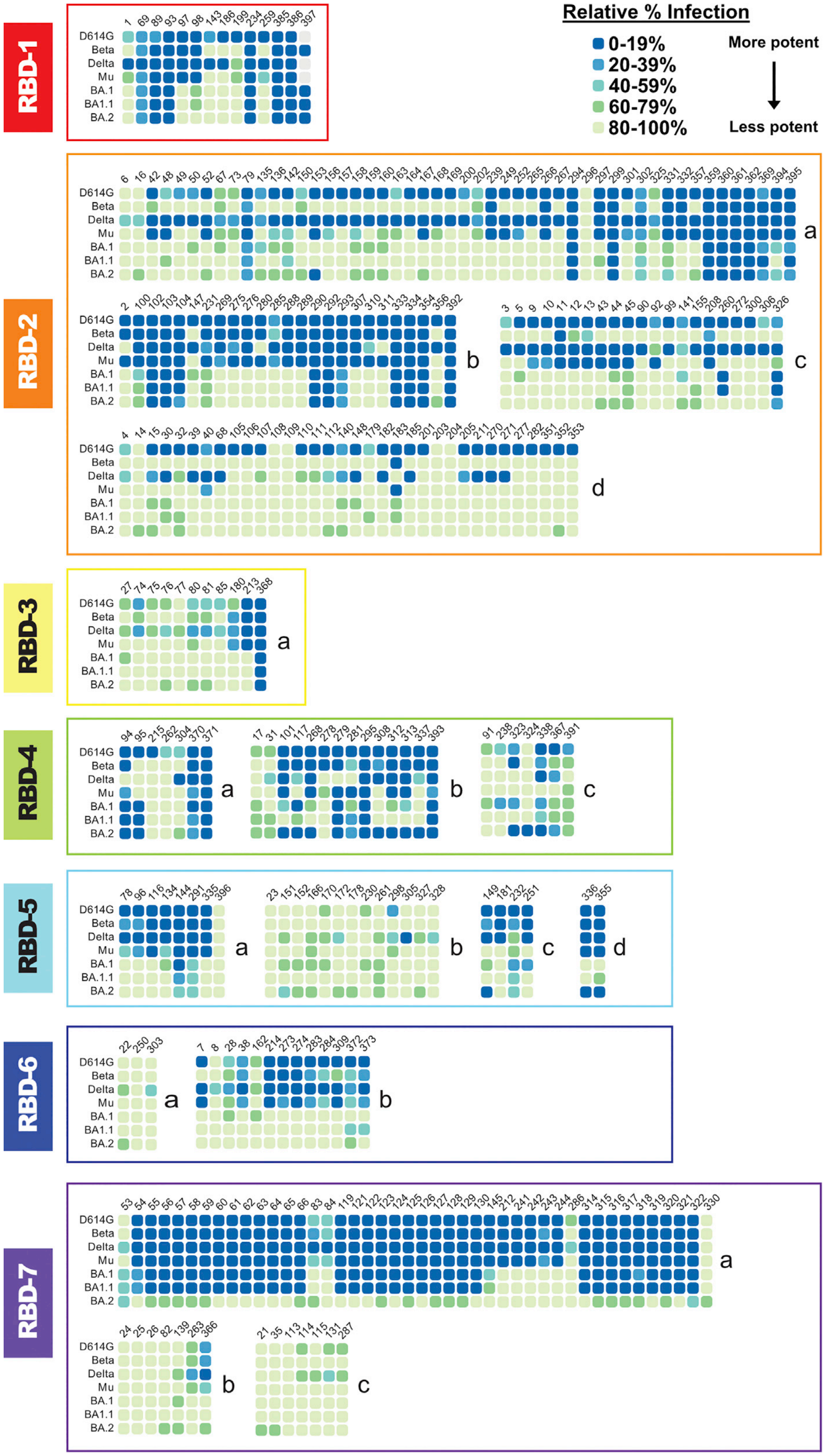

(legend on next page)

determine neutralization titer. We then examined how retention of Omicron neutralization relates to competition group and antibody structure, as well as other antibody features. The results of this study provide information that can be used to guide both therapeutic selection and vaccine design.

RESULTS

We first tested 397 antibodies in the CoVIC panel series in a block neutralization assay. Antibodies were aliquoted into 96-well plates at two concentrations (25 μg/mL and 250 ng/mL) and then incubated with vesicular stomatitis virus (VSV)-based pseudovirus displaying spike protein from either D614G, the Beta (B.1.529), Delta, or Mu VoCs, or one of three Omicron lineages: BA.1 (also known as B.1.1.519), BA1.1, or BA.2. This initial analysis allowed rapid identification of antibodies that retained detectable potency against Omicron.

At 250 ng/mL, 66 antibodies in the panel (~16%) had potent neutralization against BA.1, resulting in ≤20% of infected cells relative to cells infected in the absence of antibody (Figures 2 and S2; Table S1). Another 14 (~3%) were less potent but still had detectable activity, with 20%–40% of cells infected. Of the 66 antibodies that potently neutralize BA.1, six belong to RBD-1, 17 to RBD-2, one to RBD-3, eight to RBD-4, one to RBD-5, 31 to RBD-7, and one to a trimer-specific site. The remaining antibody did not have a binding site assigned. The 14 antibodies with intermediate potency included one RBD-1, four RBD-2, three RBD-4, two RBD-5, two RBD-7, one S2 binder, and one unassigned antibody.

At the higher concentration of 25 μg/mL, an additional 67 antibodies (133 total, 34%) neutralized BA.1, with ≤20% cells infected (Table S1). Ten of these antibodies belong to the RBD-1 epitope group, 32 to RBD-2, one to RBD-3, 18 to RBD-4, 10 to RBD-5, three to RBD-6, and 38 to RBD-7. Two are S2 binders, two are NTD binders, three are polyclonal antibodies, one binds trimer, and the remaining 13 antibodies do not yet have an epitope assigned.

Mutations that affect antibody neutralization

The three Omicron variants in the block neutralization assay contain 15, 16, and 16 mutations in the RBD for BA.1, BA1.1, and BA.2, respectively, compared with D614G spike. The BA.1 and BA1.1 examined here differ by a single residue at 346 and have similar neutralization profiles (Figures 1 and 2). BA.2 shares many of its mutations with BA.1 but has additional mutations at residues 376, 405, and 408 and lacks mutations at residues 446 and 496 (Figure 1). As a result, BA.2 shows a substantially different neutralization profile than BA.1 and BA1.1 (Figure 2). Notably, antibodies in the RBD-7 community neutralize BA.1 and BA1.1 well but do not neutralize BA.2 (Figure 2). Conversely, several RBD-4 and -5 community members neutralize BA.2 well but not BA.1 or BA1.1 (Figure 2). The loss of BA.2 neutralization in the RBD-7 community is likely due to the T376A and/or D405N point mutations in the RBD-7 antigenic site, while some antibodies in the RBD-4 and -5 communities likely regain neutralization because BA.2 lacks G446S, G496S, and R346K mutations, which overlap the RBD-4 and -5 antigenic sites (Figure 1).

Examining antibody neutralization profiles and RBD differences between SARS-CoV-2 variants yields additional insight into how mutations affect antibody neutralization. For example, multiple members of the RBD-2c epitope community neutralize D614G, Delta, and Mu strains well but not Beta. The only RBD mutation unique to Beta, K417N, lies within the RBD-2 epitope and likely disrupts binding by these antibodies. Similarly, we can conclude that many members of the RBD-1 and -2a communities are sensitive to mutations at 484 and 501 (and possibly 417).

BA.4/5 and BA.2.12.1 neutralization

We further tested the neutralization activity of 20 antibodies that potently neutralized D614G, Beta, Delta, Mu, BA.1, BA1.1, and BA.2 against pseudovirus bearing BA.4/5 and BA2.12.1 spike. Although neutralization titers were lower against BA2.12.1 compared with the parent D614G strain for multiple antibodies, all maintained potent neutralization against BA.2.12.1 (Figure S3). For BA.4/5, however, six antibodies lost neutralization and another eight could not completely neutralize virus at the highest antibody concentration tested (Figure S3). This loss of activity is likely due to the F486V RBD mutation, which is unique to the BA.4 and BA.5 sublineages. The remaining seven antibodies were unaffected by the F486V mutation and neutralized BA.4/5 pseudovirus as well as or better than the D614G parent strain (Figure S3).

Bivalent binding

Structural biology has traditionally studied the antigen-binding fragment (Fab) and considered single-Fab footprints when evaluating breadth of neutralization. Here, we evaluated intact IgG, the biologically relevant form that protects after infection, vaccination, or prophylactic or therapeutic delivery of antibodies. We examined the binding interactions between Omicron-neutralizing IgG and spike using negative-stain electron microscopy. We chose 18 antibodies that neutralize Omicron well and cover a representative range of the Omicron-neutralizing epitope groups, with two RBD-1 community members, ten RBD-2, one RBD-3, three RBD-4, one RBD-5, and one RBD-7. RBD-2 is the most abundant epitope group in the experiment and was also the most common epitope targeted by the therapeutic candidates. Although RBD-7 contains multiple antibodies that retain Omicron neutralization, most members of the RBD-7 group are not natural IgGs but instead are engineered, tetravalent assemblies of heavy chain-only nanobody (VHH) or scFv domains. When incubated with spike ectodomains, these molecules form large aggregates that are unsuitable for structural analysis.

Figure 2. Block neutralization assay of CoVIC antibodies against pseudovirus displaying spike from SARS-CoV-2 VoCs
Results for neutralization of SARS-CoV-2 pseudovirus by 250 ng/mL antibody are shown. Antibodies are organized by antigenic competition group and subgroup. Neutralization activity is expressed as the percentage of infected cells in the presence of antibody relative to cells infected in the absence of antibody. See also Figures S2 and S3 and Table S1.

For the RBD-7 sample selected, CoVIC-63, we first digested the molecule and purified the individual VHH domains before forming complexes with spike in order to prevent formation of cross-linked aggregates.[15]

We found that 13 of the 18 Omicron-neutralizing antibodies (~70%), including all of the RBD-2 binding antibodies, bind spike bivalently, with each arm of the antibody engaging a neighboring RBD in spike (Figure 3A). All of the RBD-2 bivalent antibodies bound two RBDs in the "up" conformation, while the RBD-3, -4, and -5 bivalent antibodies each bound two RBDs in the "down" conformation. Of the remaining five non-bivalent, Omicron-neutralizing antibodies, two are ACE-2-Fc constructs and one is a multivalent RBD-7, leaving the two RBD-4b binders as the only natural antibodies that bind monovalently and neutralize Omicron. These RBD-4b binders likely maintain Omicron neutralization despite their monovalent interaction because the RBD-4 footprint has greater sequence conservation between D614G and the Omicron variants. In the RBD-4 footprint, there are only three Omicron substitutions: R346K, G446S, and Q498R. Each is either a biochemically conserved mutation (346 and 446) or at the edge of the binding footprint (498) (Figure 1).

Our previous electron microscopy work with spike-binding antibodies in CoVIC showed that antibodies from different epitope communities approach the RBD from different angles. The approach angle and resulting position of the rest of the IgG determine whether the antibody binds to spike monovalently, bivalently within one spike (intra-spike crosslinking), or by connecting two different spikes (inter-spike crosslinking).[15] In particular, many RBD-2 and some RBD-5 community members tend to bind bivalently, with intra-spike crosslinking. The remaining epitope communities typically bind monovalently (one Fab to one spike, with the other Fab free) or crosslink separate spike trimers (each Fab binds a different spike).

We hypothesized that bivalent binding, which would increase the strength of interaction between an antibody and a spike protein, may allow these antibodies to retain neutralization activity toward Omicron despite its many mutations. To test this hypothesis, we first used negative-stain electron microscopy to determine the extent to which bivalency is linked to Omicron neutralization. In this analysis, we selected an additional nine antibodies from the RBD-2 community that failed to neutralize Omicron. For these antibodies, eight of the nine bind entirely or partially monovalently, with 22%–100% of the antibody/spike complexes comprising monovalently bound spikes (Figure 3B). In contrast, each RBD-2 antibody that does neutralize Omicron exhibits only bivalent binding, with no monovalent binding interactions visible in any 2D class (Figure 3A).

Impact of bivalency on neutralization

To further test the importance of bivalent binding for Omicron neutralization, we isolated Fab fragments from a subset of the Omicron-neutralizing antibodies and compared the D614G and BA.1 pseudovirus neutralization activity of Fabs with that of the corresponding intact IgG. In this analysis, we included six RBD-2 bivalent binders, as well as one RBD-3 bivalent binder and one RBD-4 monovalent binder as controls. All eight IgGs neutralize D614G pseudovirus well (Figure 4). Five of the eight IgGs neutralize BA.1 and D614G equally well, while the remaining three (CoVIC-292, -334, and -260) have a 6- to 24-fold reduction in neutralization activity for BA.1 pseudovirus compared with D614G (Figure 4).

Against D614G pseudovirus, all Fabs have lower neutralization activity compared with intact IgG (Figure 4), suggesting, as expected, that inter/intra-spike crosslinking or steric hindrance imposed by the larger mass of the IgG molecule can inhibit viral infection. However, digestion of the bivalently binding IgGs into Fabs disproportionately decreases neutralization of BA.1 pseudovirus compared with the monovalent binding control CoVIC-295 (Figure 4). While CoVIC-295 Fabs neutralize D614G and BA.1 pseudovirus equally (albeit to lower levels than CoVIC-295 IgGs) (Figure 4), Fabs from bivalent-binding antibodies all neutralize BA.1 pseudovirus worse than D614G pseudovirus, and some fail to neutralize BA.1 pseudovirus at all. This effect is particularly pronounced for CoVIC-290, -333, and -361. For these antibodies, the IgGs neutralize both D614G and BA.1 with essentially equal IC_{50} values, but the Fab fragments exhibit either a 20-fold reduction in neutralization relative to D614G or do not neutralize BA.1 at all (Figure 4).

Function of bivalent binding

To confirm that the disproportionate loss of Omicron pseudovirus neutralization is due to loss of avidity after IgGs are digested into Fabs, rather than to crosslinking or steric hindrance, we used surface plasmon resonance (SPR) to measure the binding kinetics between BA.1 spike trimers and six sets of IgGs and the corresponding Fabs. We also measured binding affinity between D614G spike and IgGs. Five sets of IgGs/Fabs (CoVIC-290, 292, -333, -334, and -368) bind bivalently to the RBD-2b or -3 epitopes. The sixth set (CoVIC-295) serves as a control and binds monovalently to RBD-4b, a footprint that has a higher degree of sequence conservation than other epitopes.

As IgG, all six antibodies bind to D614G spike with very high (K_D between 10^{-10} and 10^{-11} M) affinity (Figure S4). Binding affinity to Omicron (BA.1) spike varies, ranging from tens of nanomolars to subnanomolars (Figure 5A). Two bivalent RBD-2 IgGs (CoVIC-290 and -292) exhibit high off-rates from BA.1 spike, resulting in lower-affinity interactions, while the remaining four IgGs disassociate more slowly and have subnanomolar affinities (Figure 5A). One bivalent RBD-2 antibody, CoVIC-333, and the monovalent RBD-4 IgG, CoVIC-295, retain high-affinity binding to BA.1 spike as Fabs (Figures 5A and 5B). The remaining three bivalent RBD-2 antibodies (CoVIC-290, -292, and -334) bind to BA.1 spike with affinities in the tens of nanomolars when digested into Fabs, and CoVIC-368, the RBD-3 bivalent binder, has nanomolar affinity for BA.1 as a Fab (Figure 5B).

Retention of binding through avidity does translate to neutralization activity. Because Fabs from CoVIC-290, -292, and -334 bind BA.1 spike poorly (Figure 5B), the Fabs fail to neutralize BA.1 pseudovirus (>10,000 ng/mL IC_{50}; Figure 4). Their bivalent IgGs, in contrast, which bind BA.1 spike with higher affinity, potently neutralize Omicron pseudovirus with IC_{50} values of 25, 73, and 70 ng/mL for CoVIC-290, -292, and -334, respectively (Figures 4 and 5B). However, the benefits of bivalent binding go beyond increased binding affinity. When we compare CoVIC-333 and -295, which bind BA.1 spike strongly both as IgG and as Fab, we note a difference in neutralization outcome

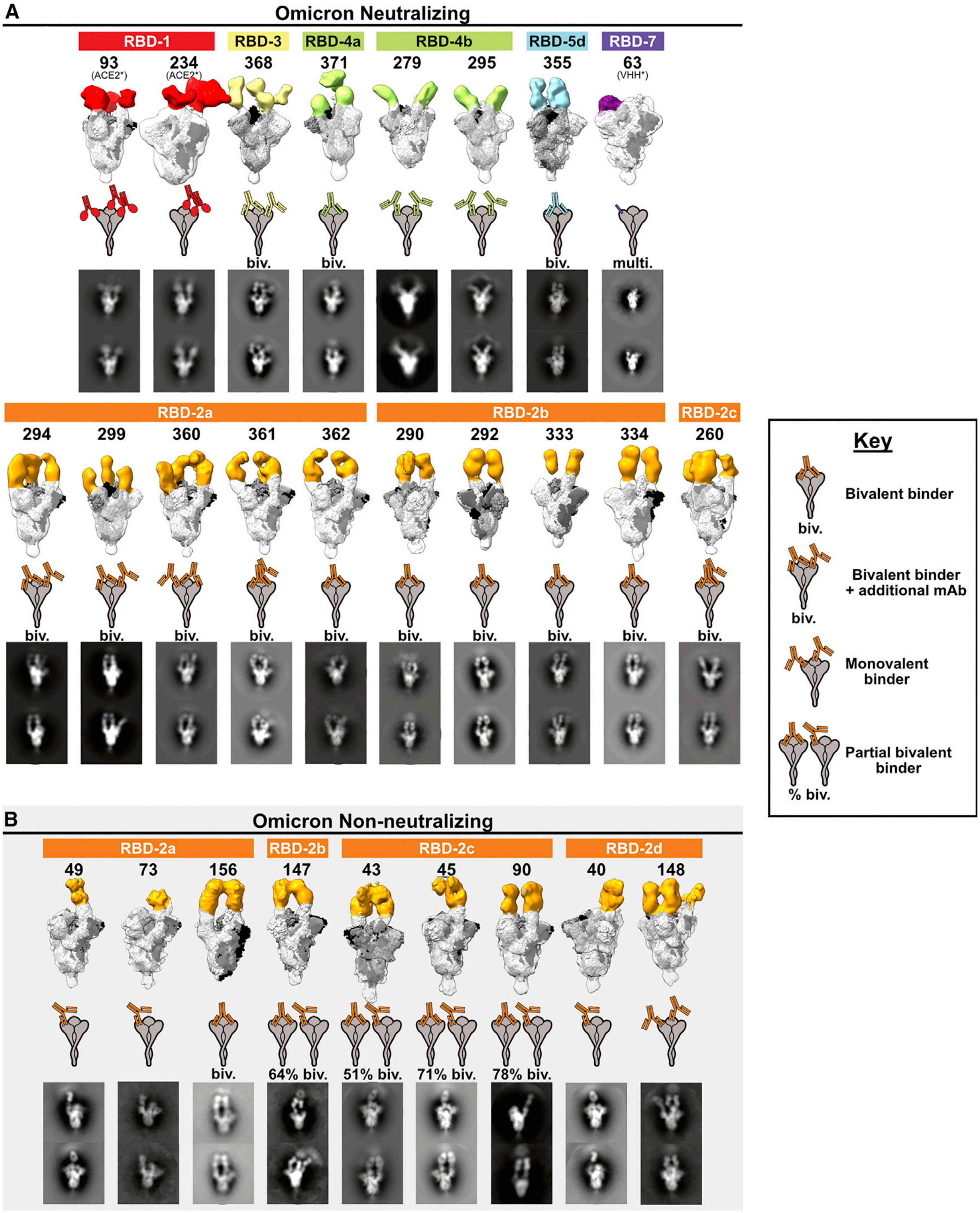

Figure 3. Negative-stain electron microscopy reconstructions of Omicron-neutralizing and non-neutralizing CoVIC antibodies

Negative-stain electron microscopy (nsEM) of BA.1 Omicron neutralizing antibodies (A) and non-neutralizing antibodies (B) in complex with HexaPro D614G spike. (Top) For each antibody, the 3D nsEM volume is shown with antibody shading that corresponds to the color scheme for the RBD antigenic site. The spike (docked model PDB: 7A94) has subunits colored black, white, and gray. (Middle) Cartoon illustrations of IgG binding patterns on spike proteins. (Bottom) Representative 2D classes showing CoVIC antibodies binding bivalently or monovalently to spike. "Biv." indicates that antibodies bind bivalently across two protomers on a single spike molecule. Some groups (e.g., RBD-3 and -2a) exhibit one bivalent IgG bound to two of the three RBDs and have an additional IgG occupying the third RBD on the trimeric spike. "Multi." indicates that the molecule is multivalent and is engineered to contain more than the two binding domains that are typical of an antibody. For antibodies that have mixed bivalent and monovalent binding to spike protein, the approximate percentage of particles that bind bivalently is shown.

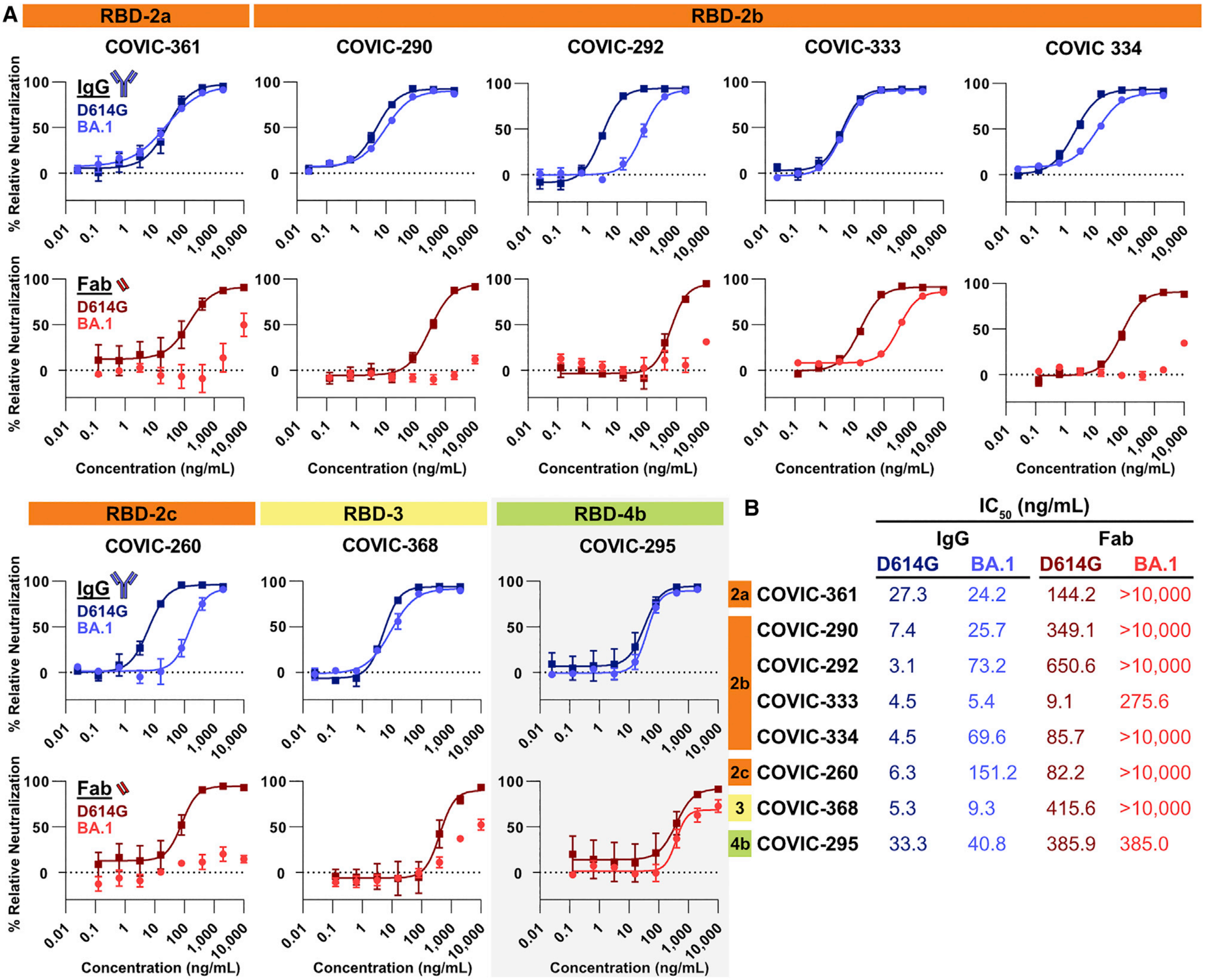

B

	IC50 (ng/mL)			
	IgG		Fab	
	D614G	BA.1	D614G	BA.1
2a COVIC-361	27.3	24.2	144.2	>10,000
2b COVIC-290	7.4	25.7	349.1	>10,000
2b COVIC-292	3.1	73.2	650.6	>10,000
2b COVIC-333	4.5	5.4	9.1	275.6
2b COVIC-334	4.5	69.6	85.7	>10,000
2c COVIC-260	6.3	151.2	82.2	>10,000
3 COVIC-368	5.3	9.3	415.6	>10,000
4b COVIC-295	33.3	40.8	385.9	385.0

Figure 4. D614G and BA.1 pseudovirus neutralization with IgG and Fabs

(A) Representative BA.1-neutralizing antibodies were tested for neutralization of pseudovirus bearing D614G or BA.1 spike pseudovirus using whole IgG molecules or Fab fragments. Curves represent 3 biological replicates. The RBD-4b mAb (gray background) is a monovalent binder; all other IgGs shown here bind bivalently. Error bars are ± SEM.

(B) IC_{50} values for CoVIC antibodies and Fabs with D614G and BA.1 pseudoviruses.

See also Figure S3.

between the Fab fragment of the bivalent binder (CoVIC-333) and the Fab fragment of the monovalent binder (CoVIC-295). Fab fragments from both antibodies bind BA.1 with high affinity and to similar levels (~1.5-fold affinity difference) (Figure 5B), but Fabs from the monovalent binder CoVIC-295 neutralize BA.1 and D614G pseudovirus equally (Figure 4), and Fabs from the bivalent binder CoVIC-333 neutralize BA.1 pseudovirus neutralization much more poorly than D614G (Figure 4). However, CoVIC-333 IgGs neutralize D614G and Omicron pseudoviruses equally well (Figure 4). Thus, bivalent binding not only helps low-affinity Fabs to retain binding as IgG but also appears to enhance the neutralization potency of high-affinity Fabs by crosslinking spike protomers or otherwise mechanically neutralizing virus.

DISCUSSION

Since its emergence in late 2019, SARS-CoV-2 has acquired numerous mutations throughout its genome, both to its spike protein and elsewhere, that affect its infectivity and ability to evade the host immune response. These mutations contributed to waves of variants that perpetuated the pandemic. Although the Omicron variant and its sublineages are the most recent and extreme example, prior VoCs including Beta, Delta, and Mu each introduced a number of mutations that affected affinity and neutralization. Recombination between SARS-CoV-2 strains is also possible, resulting in hybrid viruses that have combinations of previously observed mutations. SARS-CoV-2 will likely continue to mutate as it adapts to humans and other host

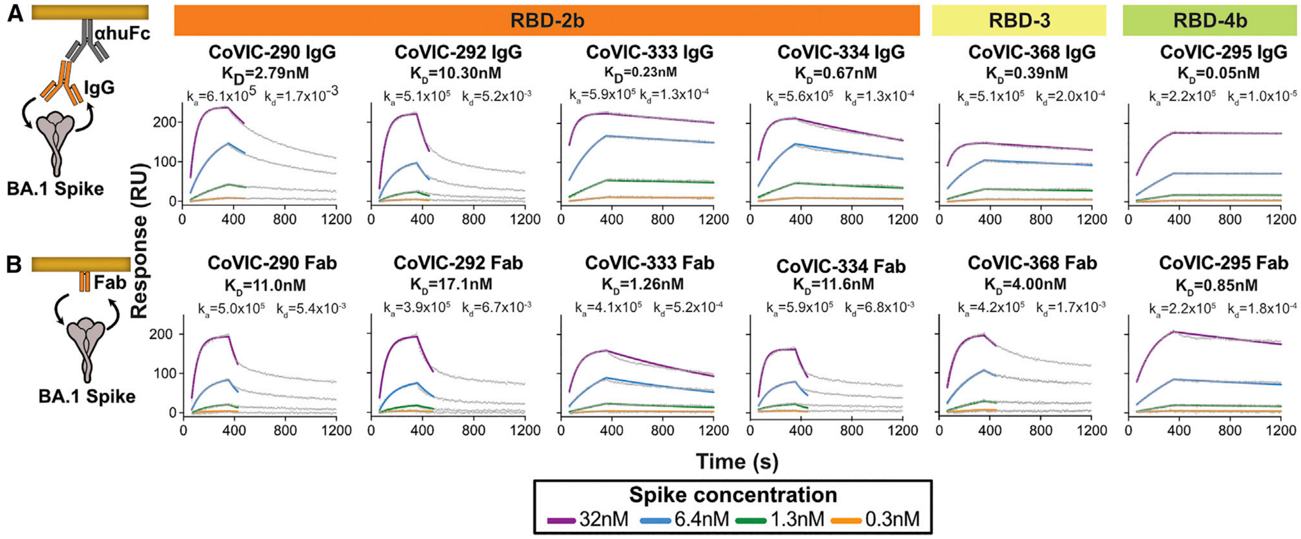

Figure 5. Binding kinetics of BA.1 spike ectodomains to IgG and Fabs

Surface plasmon resonance experiments comparing the binding kinetics of IgG (A) and Fab (B) to Omicron BA.1 spike. For each sample, experimental data (gray line) and 1:1 fitted curve (colored lines) are shown. Spike concentrations range from 0.3–32 nM. K_D, k_a, and k_d values for each interaction are indicated. See also Figure S4.

species and undergoes selective pressure in each species, as well as against vaccine responses and antiviral therapeutics.

In this work, we analyzed nearly 400 antibody therapeutic candidates to learn the determinants for durable neutralization against Omicron and its sublineages, which have the greatest divergence of all the VoCs relative to the original strains. This study focused on antibodies against the RBD, as antibodies against this site exhibit the most potent neutralization, these epitopes are immunodominant, and the majority of therapeutic candidates target this domain. Epitopes in the fusion subunit S2 are more conserved but rarely neutralize as potently.

The mutations in BA.1, BA1.1, and BA.2 spikes each sharply decrease the number of antibodies capable of neutralizing these variants at therapeutically relevant concentrations. We show that mutations in Omicron affect antibodies in every RBD epitope community, as these mutations span the binding footprints of all seven epitope communities. Despite the large number of mutations, however, over 16% of the study panel maintain durable neutralization of Omicron, Beta, Delta, and Mu VoCs.

Using electron microscopy to analyze IgG interactions with spike and SPR to measure IgG and Fab binding affinity, we found that IgGs that retain robust neutralization of Omicron and other VoCs tend to bind bivalently. These antibodies attach to spike with both Fabs simultaneously, with both spike RBDs in either the up or down conformation depending on the antibody. In contrast, most monovalently binding IgGs within the same epitope group lose binding and neutralization against Omicron. Bivalent binding compensates for mutation-decreased binding affinity of each Fab to variant spike with avidity, effectively squaring the binding capacity of an individual Fab for the spike protein. When we remove the capacity for bivalent binding by digesting antibodies into Fabs, many of these antibodies lose their ability to neutralize Omicron, even when the parent IgG molecule neutralizes Omicron potently, and both Fab and IgG neutralize D614G. In short, emergence of mutations leads to loss of antibody neutralization unless the antibody recognizes a conserved epitope or can compensate for mutations with avidity, and bivalent binding may further enhance neutralization by crosslinking spike protomers or otherwise inactivating spike.

These results suggest that bivalently binding IgGs could be prioritized as therapeutics or that bi- or multivalency could be engineered into therapeutic molecules. These results further suggest that spike-based vaccine strategies, in which RBDs are displayed in the appropriate distance and orientation to elicit bivalent IgG recognition, could lead to more durable, variant-resistant antibody responses. Similarly, antigen density on nanoparticle vaccines that mimics that on the virus would facilitate elicitation of durability via intra-spike bivalent binding. RBD-based vaccines, such as on nanoparticles, would benefit from ensuring spike-like spacing. Further engineering of spike protein to make the RBD conformations homogeneous (all up or all down) may further favor the generation of antibodies that can use avidity to their advantage.

Limitations of the study

Most of the antibodies examined in this study were generated relatively early in the COVID-19 pandemic, before the emergence of Omicron and many of the other VoCs. Individuals who were infected by the later VoCs or who have received Omicron booster vaccines may have developed broadly neutralizing antibodies that can better target both the original SARS-CoV-2 strain and the VoCs. It is possible that these antibodies, if they exist, neutralize virus by targeting different antigenic sites or via different mechanisms than those described in this work. The antibodies selected for this study were also selected for the ability to neutralize virus *in vitro*. There may also be

antibodies that protect against Omicron or other VoCs *in vivo* but do not neutralize *in vitro*, and they could have different mechanisms of action from those described here.

STAR★METHODS

Detailed methods are provided in the online version of this paper and include the following:

- KEY RESOURCES TABLE
- RESOURCE AVAILABILITY
  - Lead contact
  - Materials availability
  - Data and code availability
- EXPERIMENTAL MODEL AND SUBJECT DETAILS
  - CoVIC antibodies
  - SARS-CoV-2 strains and spike proteins
  - SARS-CoV-2 pseudovirus
- METHOD DETAILS
  - Standard and block neutralization assays
  - Antibody digestion and Fab purification
  - Negative stain electron microscopy
  - Surface plasmon resonance
- QUANTIFICATION AND STATISTICAL ANALYSIS

SUPPLEMENTAL INFORMATION

Supplemental information can be found online at https://doi.org/10.1016/j.celrep.2023.112014.

ACKNOWLEDGMENTS

We thank Dr. Ruben Dias Avalos and the LJI Cryoelectron Microscopy Facility for data collection at LJI and Milite Abraha and Gillian Q. Horn for data collection at Duke. The development and analysis of one set of antibodies was supported by the Division of Intramural Research, National Institute of Allergy and Infectious Diseases (NIAID), National Institutes of Health (NIH). We thank the Overton family for launching the study of variants for the CoVIC. The authors are indebted to the many contributors of samples to the CoVIC study. Without their generosity and contributions, a study on this scale would not have been possible. We are thankful for INV-0006133 (E.O.S. and B.P.) and INV-008612 (G.T.) of the Bill and Melinda Gates Foundation, NIH U19 AI142790-03S1, and the GHR Foundation (E.O.S.) for support of this study. We also acknowledge funding from NIH/NIAID grants R44AI145491 and R44AI134269 (M.D.A., C.C.B., and J.M.T.). L.Y. was supported by project #202201020528 of Guangzhou Municipal Science and Technology Bureau. S.K. acknowledges funding from the US Department of Defense, Defense Health Agency, through the CARES Act. Additional funding was executed through a cooperative agreement (W81XWH-18-2-0040) between the Medical Research and Development Command of the Army Futures Command in the US Department of the Army and the Henry M. Jackson Foundation. The opinions or assertions contained herein are the private views of the authors and are not to be construed as official or as reflecting true views of the Department of the Army or the Department of Defense. M.G. and N.I. acknowledge support from KU Leuven KOOR ZKD8270, KU Leuven SARS-CoV-2 mAb OF2, and KU Leuven C3/20/105. Isolation of one set of antibodies was supported by the Emergency Project for COVID-19 from Chongqing Medical University and Chongqing Science and Technology Commission (cstc2021jscx-fyzxX0001) of China.

AUTHOR CONTRIBUTIONS

Conceptualization, H.M.C., K.M.H., S.L.S., and E.O.S.; methodology, H.M.C. and K.M.H.; validation, H.M.C. and K.M.H.; formal analysis, H.M.C. and K.M.H.; investigation, H.M.C., K.M.H., H.L., X.Y., J.S., T.B., S.H., S.M.D., K.L., G.T., D.B., and C.T.; resources, M.A., C.C.B., S.B., J.L.B., J.-Q.C., C.C., H.C., P.D.C., V.D., K.C.E., Y.E., J.K.F., N.G., A.E.G., Y.G., X.H., C.J.H., J.M.H., B.H., C.H., A.H., M.I., A.J., N.K., G.K., M.K., J.K.K., S.J.K., T.L., F.L., T.M., M.A.M., L.M.-R., A.M., C.J.O., B.E.R.R., A.K.S., M.S., A.S., S.S., J.T., J.M.T., D.P.U., Y.W., L.Y., T.Z.Y., and E.Y.; data curation, B.P., S.L.S., K.M.H., D.B., and C.T.; writing – original draft, H.M.C., S.L.S., and E.O.S.; writing – review & editing, H.M.C., K.M.H., S.L.S., and E.O.S.; visualization, H.M.C. and K.M.H.; supervision, E.O.S.; project administration, S.L.S.; funding acquisition, E.O.S., B.P., G.T., M.D.A., C.C.B., J.M.T., L.Y., S.J.K., N.G., and M.I.

DECLARATION OF INTERESTS

C.C.B. and M.D.A. are inventors on the patent application PCT/US21/33454, which covers the version of ACE2-Ig used in this study. The patent application is assigned to Emmune, Inc. M.D.A., C.C.B., and J.M.T. own stock in Emmune, Inc. Y.G. is co-founder and shareholder in Antibody BioPharm, Inc., and ChangYuan FuNeng (Shanghai) Life Technology Co., Ltd. L.Y. and Y.G. have filed patent applications on related COVID-19 antibodies. P.D.C., J.T., and H.C. are inventors on several of the antibodies described in this study.

INCLUSION AND DIVERSITY

We support inclusive, diverse, and equitable conduct of research.

Received: October 17, 2022
Revised: November 25, 2022
Accepted: January 5, 2023

REFERENCES

1. WHO Coronavirus (COVID-19) Dashboard https://covid19.who.int/.
2. CDC (2020). COVID Data Tracker (Centers for Disease Control and Prevention). https://covid.cdc.gov/covid-data-tracker/.
3. Covid-19 Vaccines. https://www.who.int/teams/regulation-prequalification/eul/covid-19.
4. Office of the commissioner Coronavirus (COVID-19) update: FDA authorizes moderna, pfizer-BioNTech bivalent COVID-19 vaccines for use as a booster dose. U.S. Food and Drug Administration. https://www.fda.gov/news-events/press-announcements/coronavirus-covid-19-update-fda-authorizes-moderna-pfizer-biontech-bivalent-covid-19-vaccines-use.
5. Viana, R., Moyo, S., Amoako, D.G., Tegally, H., Scheepers, C., Althaus, C.L., Anyaneji, U.J., Bester, P.A., Boni, M.F., Chand, M., et al. (2022). Rapid epidemic expansion of the SARS-CoV-2 Omicron variant in southern Africa. Nature *603*, 679–686. https://doi.org/10.1038/s41586-022-04411-y.
6. Update on Omicron https://www.who.int/news/item/28-11-2021-update-on-omicron.
7. Han, P., Li, L., Liu, S., Wang, Q., Zhang, D., Xu, Z., Han, P., Li, X., Peng, Q., Su, C., et al. (2022). Receptor binding and complex structures of human ACE2 to spike RBD from omicron and delta SARS-CoV-2. Cell *185*, 630–640.e10.
8. Zahradník, J., Marciano, S., Shemesh, M., Zoler, E., Harari, D., Chiaravalli, J., Meyer, B., Rudich, Y., Li, C., Marton, I., et al. (2021). SARS-CoV-2 variant prediction and antiviral drug design are enabled by RBD in vitro evolution. Nat. Microbiol. *6*, 1188–1198.
9. Tegally, H., Wilkinson, E., Giovanetti, M., Iranzadeh, A., Fonseca, V., Giandhari, J., Doolabh, D., Pillay, S., San, E.J., Msomi, N., et al. (2021). Detection of a SARS-CoV-2 variant of concern in South Africa. Nature *592*, 438–443.
10. Netzl, A., Tureli, S., LeGresley, E., Mühlemann, B., Wilks, S.H., and Smith, D.J. (2022). Analysis of SARS-CoV-2 omicron neutralization data up to 2021-12-22. Preprint at bioRxiv. https://doi.org/10.1101/2021.12.31.474032.

11. Tada, T., Zhou, H., Samanovic, M.I., Dcosta, B.M., Cornelius, A., Mulligan, M.J., Landau, N.R., Mulligan, M.J., and Landau, N.R. (2021). Increased resistance of SARS-CoV-2 omicron variant to neutralization by vaccine-elicited and therapeutic antibodies. Preprint at bioRxiv. https://doi.org/10.1101/2021.12.28.474369.

12. Lusvarghi, S., Pollett, S.D., Neerukonda, S.N., Wang, W., Wang, R., Vassell, R., Epsi, N.J., Fries, A.C., Agan, B.K., Lindholm, D.A., et al. (2021). SARS-CoV-2 Omicron neutralization by therapeutic antibodies, convalescent sera, and post-mRNA vaccine booster. Preprint at bioRxiv. https://doi.org/10.1101/2021.12.22.473880.

13. Walls, A.C., Sprouse, K.R., Bowen, J.E., Joshi, A., Franko, N., Navarro, M.J., Stewart, C., Cameroni, E., McCallum, M., Goecker, E.A., et al. (2022). SARS-CoV-2 breakthrough infections elicit potent, broad, and durable neutralizing antibody responses. Cell *185*, 872–880.e3. https://doi.org/10.1016/j.cell.2022.01.011.

14. Andrews, N., Stowe, J., Kirsebom, F., Toffa, S., Rickeard, T., Gallagher, E., Gower, C., Kall, M., Groves, N., O'Connell, A.-M., et al. (2022). Covid-19 vaccine effectiveness against the omicron (B.1.1.529) variant. N. Engl. J. Med. *386*, 1532–1546.

15. Hastie, K.M., Li, H., Bedinger, D., Schendel, S.L., Dennison, S.M., Li, K., Rayaprolu, V., Yu, X., Mann, C., Zandonatti, M., et al. (2021). Defining variant-resistant epitopes targeted by SARS-CoV-2 antibodies: a global consortium study. Science *374*, 472–478.

16. Barnes, C.O., Jette, C.A., Abernathy, M.E., Dam, K.-M.A., Esswein, S.R., Gristick, H.B., Malyutin, A.G., Sharaf, N.G., Huey-Tubman, K.E., Lee, Y.E., et al. (2020). SARS-CoV-2 neutralizing antibody structures inform therapeutic strategies. Nature *588*, 682–687.

17. Cho, H., Gonzales-Wartz, K.K., Huang, D., Yuan, M., Peterson, M., Liang, J., Beutler, N., Torres, J.L., Cong, Y., Postnikova, E., et al. (2021). Bispecific antibodies targeting distinct regions of the spike protein potently neutralize SARS-CoV-2 variants of concern. Sci. Transl. Med. *13*, eabj5413.

18. Dussupt, V., Sankhala, R.S., Mendez-Rivera, L., Townsley, S.M., Schmidt, F., Wieczorek, L., Lal, K.G., Donofrio, G.C., Tran, U., Jackson, N.D., et al. (2021). Low-dose in vivo protection and neutralization across SARS-CoV-2 variants by monoclonal antibody combinations. Nat. Immunol. *22*, 1503–1514.

19. Imbrechts, M., Maes, W., Ampofo, L., Van den Berghe, N., Calcoen, B., Van Looveren, D., Kerstens, W., Rasulova, M., Vercruysse, T., Noppen, S., et al. (2022). Potent neutralizing anti-SARS-CoV-2 human antibodies cure infection with SARS-CoV-2 variants in hamster model. iScience *25*, 104705.

20. Rothenberger, S., Hurdiss, D.L., Walser, M., Malvezzi, F., Mayor, J., Ryter, S., Moreno, H., Liechti, N., Bosshart, A., Iss, C., et al. (2022). The trispecific DARPin ensovibep inhibits diverse SARS-CoV-2 variants. Nat. Biotechnol. *40*, 1845–1854. https://doi.org/10.1038/s41587-022-01382-3.

21. Iwanaga, N., Cooper, L., Rong, L., Maness, N.J., Beddingfield, B., Qin, Z., Crabtree, J., Tripp, R.A., Yang, H., Blair, R., et al. (2022). ACE2-IgG1 fusions with improved in vitro and in vivo activity against SARS-CoV-2. iScience *25*, 103670.

22. Han, X., Wang, Y., Li, S., Hu, C., Li, T., Gu, C., Wang, K., Shen, M., Wang, J., Hu, J., et al. (2021). A rapid and efficient screening system for neutralizing antibodies and its application for SARS-CoV-2. Front. Immunol. *12*, 653189.

23. Snyder, T.M., Gittelman, R.M., Klinger, M., May, D.H., Osborne, E.J., Taniguchi, R., Zahid, H.J., Kaplan, I.M., Dines, J.N., Noakes, M.T., et al. (2020). Magnitude and dynamics of the T-cell response to SARS-CoV-2 infection at both individual and population levels. Preprint at medRxiv. https://doi.org/10.1101/2020.07.31.20165647.

24. Dines, J.N., Manley, T.J., Svejnoha, E., Simmons, H.M., Taniguchi, R., Klinger, M., Baldo, L., and Robins, H. (2020). The ImmuneRACE study: a prospective multicohort study of immune response action to COVID-19 events with the ImmuneCODETM open access Database. Preprint at medRxiv. https://doi.org/10.1101/2020.08.17.20175158.

25. Howie, B., Sherwood, A.M., Berkebile, A.D., Berka, J., Emerson, R.O., Williamson, D.W., Kirsch, I., Vignali, M., Rieder, M.J., Carlson, C.S., and Robins, H.S. (2015). High-throughput pairing of T cell receptor α and β sequences. Sci. Transl. Med. *7*, 301ra131.

26. Hsieh, C.-L., Goldsmith, J.A., Schaub, J.M., DiVenere, A.M., Kuo, H.-C., Javanmardi, K., Le, K.C., Wrapp, D., Lee, A.G., Liu, Y., et al. (2020). Structure-based design of prefusion-stabilized SARS-CoV-2 spikes. Science *369*, 1501–1505.

27. Pettersen, E.F., Goddard, T.D., Huang, C.C., Meng, E.C., Couch, G.S., Croll, T.I., Morris, J.H., and Ferrin, T.E. (2021). UCSF ChimeraX: structure visualization for researchers, educators, and developers. Protein Sci. *30*, 70–82.

STAR★METHODS

KEY RESOURCES TABLE

REAGENT or RESOURCE	SOURCE	IDENTIFIER
**Antibodies**		
CoVIC antibodies (CoVIC 1 to CoVIC 397)	This study and Hastie and Li et al.[15]	https://covic.lji.org
**Bacterial and virus strains**		
VSV-ΔG-GFP	Karafast	Cat# EH1020
SARS-CoV-2 D614G/Vesicular Stomatitis Virus pseudovirus	This study	N/A
SARS-CoV-2 Beta/Vesicular Stomatitis Virus pseudovirus	This study	N/A
SARS-CoV-2 Delta/Vesicular Stomatitis Virus pseudovirus	This study	N/A
SARS-CoV-2 Mu/Vesicular Stomatitis Virus pseudovirus	This study	N/A
SARS-CoV-2 Omicron BA.1/Vesicular Stomatitis Virus pseudovirus	This study	N/A
SARS-CoV-2 Omicron BA1.1/Vesicular Stomatitis Virus pseudovirus	This study	N/A
SARS-CoV-2 Omicron BA.2/Vesicular Stomatitis Virus pseudovirus	This study	N/A
SARS-CoV-2 Omicron BA2.12.1/Vesicular Stomatitis Virus pseudovirus	This study	N/A
SARS-CoV-2 Omicron BA.4/5/Vesicular Stomatitis Virus pseudovirus	This study	N/A
**Chemicals, peptides, and recombinant proteins**		
Uranyl formate	Electron Microscopy Sciences	Cat# 22451
Papain	Sigma	Cat# P3125
L-cysteine	Calbiochem	Cat# 4400
SARS-CoV-2 D614G Spike protein ectodomain with HexaPro mutations and C-terminal Foldon, HRV3C protease cleavage site, 8x-His-tag, and strep-tag	This study	N/A
SARS-CoV-2 Omicron BA.1 Spike protein ectodomain with HexaPro mutations and C-terminal Foldon, HRV3C protease cleavage site, 8x-His-tag, and strep-tag	This study	N/A
**Critical commercial assays**		
ExpiFectamine CHO Transfection kit	ThermoFisher	Cat# A29129
**Deposited data**		
Negative stain electron microscopy reconstructions of antibodies in complex with SARS-CoV-2 D614G spike	Electron Microscopy Data Bank (http://www.emdataresource.org/)	EMD: 24340, 24341, 24342, 24344, 24353, 24357, 24358, 28090, 28091, 28092, 28093, 28094, 28095, 28096, 28097, 28098, 28099, 28100, 28102, 28103, 28104, 28105, 28106, 28168, 28169, 28170, 28171
Table containing neutralization activity of all items in the CoVIC panel against pseudovirus bearing major SARS-CoV-2 VoCs	Mendeley Data (https://data.mendeley.com/)	Mendeley Data: https://dx.doi.org/10.17632/xjgsyxckt3.1
**Experimental models: Cell lines**		
293T cells	ATCC	Cat# CRL-3216; RRID:CVCL_0063
Vero cells	ATCC	Cat# CRL-1586; RRID:CVCL_0574
ExpiCho-S cells	Thermo Fisher Scientific	Cat# A29127; RRID:CVCL_5J31

(*Continued on next page*)

Continued

REAGENT or RESOURCE	SOURCE	IDENTIFIER
Recombinant DNA		
Empty vector: phCMV3	Genlantis	Cat# P003300
pCAGGS-VSV-G	Kerfast	Cat# EH1017
phCMV3-D614G Spike	This study	Genbank: QHD43416.1 with D614G mutation
phCMV3-Beta Spike	This study	Genbank: QHD43416.1 with L18F, D80A, D215G, Δ242-244, R246I, K417N, E484K, N501Y, D614G, and A701V mutations
phCMV3-Delta Spike	This study	Genbank: QHD43416.1 with T19R, G142D, E156G, Δ157–158, L452R, T478K, D614G, P681R, and D950N mutations
phCMV3-Mu Spike	This study	Genbank: QHD43416.1 with T95I, Y144S, Y145N, N146ins, R346K, E484K, N501Y, D614G, P681H, and D950N mutations
phCMV3-Omicron BA.1 Spike	This study	Genbank: QHD43416.1 with A67V, Δ69/70, T95I, G142D, Δ143/145, N211I, Δ212, ins214 EPE, G339D, S371L, S373P, S375F, S477N, T478K, E484A, Q493R, G496S, Q498R, N501Y, Y505H, T547K, D614G, H655Y, N679K, P681H, D796Y, N856K, Q954H, N969K, and L981F mutations
phCMV3-Omicron BA1.1 Spike	This study	Genbank: QHD43416.1 with A67V, Δ69/70, T95I, G142D, Δ143/145, N211I, Δ212, ins214 EPE, G339D, R346K, S371L, S373P, S375F, S477N, T478K, E484A, Q493R, G496S, Q498R, N501Y, Y505H, T547K, D614G, H655Y, N679K, P681H, D796Y, N856K, Q954H, N969K, and L981F mutations
phCMV3-Omicron BA.2 Spike	This study	Genbank: QHD43416.1 with T19I, L24S, Δ25/27, G142D, V213G, ins214 EPE, G339D, S371F, S373P, S375F, T376A, D405N, R408S, K417N, N440K, S477N, T478K, E484A, Q493R, Q498R, N501Y, Y505H, D614G, H655Y, N679K, P681H, N764K, D796Y, Q954H, and N969K mutations
phCMV3-Omicron BA.2.12.1 Spike	This study	Genbank: QHD43416.1 with T19I, L24S, Δ25/27, G142D, V213G, ins214 EPE, G339D, S371F, S373P, S375F, T376A, D405N, R408S, K417N, N440K, L452Q, S477N, T478K, E484A, Q493R, Q498R, N501Y, Y505H, D614G, H655Y, N679K, P681H, S740L, N764K, D796Y, Q954H, and N969K mutations
phCMV3-Omicron BA.4/5 Spike	This study	Genbank: QHD43416.1 with T19I, L24S, Δ25/27, Δ69/70, G142D, V213G, ins214 EPE, G339D, S371F, S373P, S375F, T376A, D405N, R408S, K417N, N440K, L452R, S477N, T478K, E484A, F486V, Q493, Q498R, N501Y, Y505H, D614G, H655Y, N679K, P681H, N764K, D796Y, Q954H, and N969K mutations
Software and algorithms		
CryoSPARC	CryoSPARC	https://www.cryosparc.com/
GraphPad Prism 9	GraphPad Software	https://www.graphpad.com/
Carterra “Kinetics” and “Epitope” software packages	Carterra	https://carterra-bio.com/
Other		
Titan Halo electron microscope	Thermo Fisher Scientific	https://www.thermofisher.com/us/en/home.html
AmMag Ni magnetic beads	GenScript	Cat# L00776
Superose 6 Increase 10/300 GL	GE Healthcare	Cat# 29091596
CellInsight CX5 High Content Screening Platform	Thermo Fisher Scientific	Cat# CX51110
CF400-Cu grids	Electron Microscopy Sciences	Cat# CF400-Cu

RESOURCE AVAILABILITY

Lead contact

Correspondence and requests for materials should be addressed to Erica Ollman Saphire (erica@lji.org).

Materials availability

Information concerning particular antibodies can be requested through the Coronavirus Immunotherapeutics Consortium at https://covic.lji.org.

Data and code availability

- Electron microscopy maps have been deposited in the Electron Microscopy Database under accession numbers EMD: 24340, 24341, 28091, 28092, 28093, 28094, 28095, 28096, 28097, 28098, 28099, 28103, 28104, 28105, 28169, 28170, 28171 and are publicly available as of the date of publication.
- This paper does not report original code.
- Additional Supplemental Items are available from Mendeley Data at https://dx.doi.org/10.17632/xjgsyxckt3.1. Any additional information required to reanalyze the data reported in this paper is available from the lead contact upon request.

EXPERIMENTAL MODEL AND SUBJECT DETAILS

CoVIC antibodies

The CoVIC antibody panel used in this study consists of 397 antibodies contributed by numerous laboratories, both industrial and academic, from five continents. The contributors to CoVIC were asked to indicate the species of origin for their antibodies. If the antibodies originated from human subjects (most were survivors of COVID-19 infection), the contributors were required to provide documentation indicating that informed consent was obtained to allow the antibodies to be used in research studies. However, the contributors were not required to provide additional information concerning patient age, gender, or other characteristics.

For fairness of the study and so that all information could be made immediately and publicly available, each antibody was assigned a number, blinding it to both experimenters and contributors. While most antibodies in the panel were isolated from individuals who had been infected by or vaccinated against SARS-CoV-2, the panel also includes bispecific, multivalent, chimeric, or otherwise engineered molecules. Several antibodies were isolated and purified as previously described.[15,17–22]

To isolate one set of antibodies, hybridoma cell lines were obtained by immunizing Balb/c mice with recombinant SARS-CoV-2 Spike protein S1 (aa1-666). Following immunization, lymphocytes were harvested from lymph nodes and immortalized by fusion with a myeloma cell. Antibodies from hybridoma cell culture supernatants were purified with Protein A or G.

The amino acid sequence of another antibody was identified from B cells of an infected individual, residues analyzed for developability liabilities, and reformatted as an IgG1 isotype as synthetic genes. A Chinese hamster ovary (CHO) stable cell pool was generated to express COVIC-355. Recombinant protein for this antibody was purified from culture supernatant using protein A and cation exchange chromatography columns, according to standard techniques.

Another set of antibodies was developed using rabbit immunization followed by humanization and affinity maturation. Rabbits were immunized with wild type RBD, and a Fab-phage library was constructed and panned against wild type spike protein trimer to isolate a highly neutralizing rabbit clone. CDRs were grafted onto a human framework to construct an initial lead candidate. A stage-based affinity maturation platform was used to further humanize the CDRs and to improve breadth of neutralization by iterative enrichment on variant spike protein trimers including wild type, Alpha, Beta, Gamma, Delta, Epsilon, and Kappa. mAbs were transiently expressed in HEK293T cells and purified by protein A chromatography.

One construct was an ACE2-Ig fusion, which was purified using a HiTrap MAbSelect SuRe Protein A column (Cytiva) that was neutralized with 1 M Tris pH 9.0. Another set of antibodies was isolated from fresh blood and frozen PBMCs obtained from previously infected donors or those with active SARS-CoV-2 infections by Bloodworks Northwest as part of the ImmuneRACE study.[23,24] Fully human antibody IgH and IgKL sequences were natively paired in high throughput using a modified version of pairSEQ[25] from isolated ASCs or memory B cells enriched for spike-specificity using flow cytometry. Abundance, isotype, and patterns of somatic hypermutation were used to select candidates for testing from the resulting set of paired sequences. Variable regions of selected antibodies were cloned into pcDNA3.4 plasmid before subcloning into an IgG1 backbone and transfection into CHO or HEK 293 cell lines for antibody production. Purity was assessed by SDS-PAGE and A280 following affinity purification.

SARS-CoV-2 strains and spike proteins

Full-length spike proteins from SARS-2-CoV strains D614G, Beta, Delta, Mu, and Omicron BA.1, BA1.1, BA.2, BA2.12.1, and BA.4/5 were cloned into the phCMV expression vector for pseudovirus production, described below.

Soluble spike ectodomains for the D614G and Omicron BA.1 strains containing the HexaPro stabilizing mutations[26] were cloned into the phCMV vector and expressed in ExpiCho cells (ThermoFisher) using the HighTiter protocol according to the manufacturer's instructions. D614G HexaPro spike protein ectodomain with C-terminal foldon, HRV3C protease, 8x His-tag, and Avi-tag was used for negative stain electron microscopy. D614G and Omicron BA.1 spike protein with C-terminal Foldon, HRV3C protease, 8x His-tag,

and double strep tag were used for Surface Plasmons Resonance (SPR) experiments. Spike proteins were purified from ExpiCho cell supernatant with AmMag Ni magnetic beads (GenScript), and then run over an S6i chromatography column (GE Healthcare).

SARS-CoV-2 pseudovirus

293T cells (ATCC CRL-3216,) in DMEM (Dulbecco's Modified Eagle Medium) (Gibco) with 10% FCS (fetal calf serum) were seeded at a concentration of 9×10^5 cells/well into 6-well plates first coated with 1:10 Poly-L-lysine (Sigma) in PBS. The following day, cells were transfected with plasmids encoding the full-length SARS-CoV-2 spike with TransIT-LT1 (Mirus Bio) according to the manufacturer's instructions. The next day, transfected cells were infected with VSV-ΔG, Vesicular Stomatitis Virus (VSV) engineered to express GFP in place of its native glycoprotein (VSV-ΔG), at a MOI of 1–2. Cells were incubated for 1 hr at 37°C in the presence of VSV-ΔG, then washed to remove unbound virus and incubated overnight in Opti-MEM (Gibco) with 2x Penicillin-Streptomycin (Gibco). At approximately 16 hpi, cell supernatant was collected and frozen at −80°C.

SARS-CoV-2 pseudoviruses were titered on Vero cells (ATCC CRL-1586) seeded in 96-well black/clear bottom plates (ThermoFisher) at a density of $2–2.5\times10^4$ cells/well. Approximately 6 hours after seeding, media was removed and 1:10 serial dilutions of pseudoviruses in Opti-MEM were added to wells. At 16 hpi, cells were fixed in 4% paraformaldehyde with 20μg/mL Hoechst for 30 min at room temperature and washed three times with PBS. Infected cells and total cells were quantitated using a Cellinsight CX5 plate reader (ThermoFisher) to determine pseudovirus titer.

METHOD DETAILS

Standard and block neutralization assays

For standard neutralization assays, IgG and Fabs were 5-fold serially diluted in Opti-MEM (Gibco) with 2x Penicillin-Streptomycin (Gibco). The IgG starting concentration was 2μg/mL and the Fab starting concentration was 10 μg/mL 7,500 fluorescence forming units (ffu) of BA.1-, BA.2.12.1-, BA.4/5- or D614G-pseudotyped VSV-ΔG in 60μL were incubated with antibody or Fab dilutions (also in 60μL, for a total of 120μL) for 1 hour at 37°C. Following incubation, 50μL of pseudovirus/antibody or pseudovirus/Fab mixtures were added to Vero cells at a density of $2–2.5\times10^4$ cells/well in 96-well black/clear bottom plates, and cells were returned to 37°C. Each antibody/pseudovirus combination was plated in duplicate. At 16 hpi, cells were fixed in 4% paraformaldehyde with 20μg/mL Hoechst for 30 min at room temperature and washed three times with PBS. Infected cells and total cells were quantitated using a Cellinsight CX5 plate reader. Percent neutralization was calculated by determining the ratio of infected cells to total cells and normalizing to wells that received VSV-ΔG without antibody. Where appropriate, IC_{50} values were calculated in GraphPad Prism 9 using non-linear regression with variable slope curve fitting. Three biological replicates were performed for each sample.

For block neutralization assays, IgG at a concentration of either 250ng/mL or 25μg/mL was used for each sample. Percent neutralization was calculated by determining the ratio of infected cells to total cells and normalizing to wells that did not receive antibody.

Antibody digestion and Fab purification

Selected CoVIC antibodies were digested into Fabs to compare neutralization and binding kinetics between Fabs and whole antibodies. Prior to digestion, papain (Sigma) was activated for 15 min at 37°C in buffer containing 100mM Tris (pH 8.0), 2mM EDTA, and 10mM L-cysteine (Sigma). 50–100μg of whole IgG were diluted in buffer containing 100mM Tris (pH 8.0) and 2mM EDTA, and incubated with 4% papain (Sigma) for 3 hours at 37°C. Following digestion, papain was inactivated with the addition of iodoacetamide to a concentration of 50mM. Cleaved IgG was incubated with Protein A agarose beads (Purolite Life Sciences) to remove the Fc (Fragment crystallizable) and any undigested IgG, and then run on an S75 FPLC column (GE Healthcare) to remove trace $F(ab')_2$. Following size exclusion chromatography, Fabs were concentrated using a 10kDa MWCO (Molecular Weight Cutoff) concentrator (Millipore).

Negative stain electron microscopy

20μg D614G HexaPro spike ectodomain was mixed with 20μg of each CoVIC antibody and incubated overnight at room temperature to form complexes. Complexes were then separated from unbound spike and antibodies via FPLC with an S6i column (GE Healthcare), and concentrated with a 100kDa MWCO concentrator (Millipore). Purified complexes were added to 400 mesh carbon grids (EMS) at a concentration of 10–20 μg/mL and incubated for 2 minutes on ice, then washed 3 times with water and stained with 0.8% uranyl formate for an additional 2 minutes on ice. Grids were imaged on an FEI Titan Halo electron microscope with either a Falcon III or K3 direct electron detector.

Image processing and reconstruction was done using cryoSPARC v3.3.1. Micrograph CTF was determined using cryoSPARC's Patch CTF Estimation algorithm. Particles were selected using either blob picker or template picker, then sorted into 2D class averages. Selected particles were then used to generate 3D reconstructions with C1 symmetry. ChimeraX[27] was used to visualize and color 3D reconstructions for publication. Percent monovalent binding was calculated by counting particles from 2D class averages.

Surface plasmon resonance

SPR measurements of binding kinetics between CoVIC antibodies and D614G and BA.1 spikes were determined as previously described in.[15] Briefly, CoVIC antibodies were either captured by goat anti-human IgG Fc antibodies coupled to an CDMP chip or

directly amine coupled to HC30M chips on the Carterra LSA platform. A five-fold dilution series of the spike was prepared in HBSTE-BSA running buffer (10 mM HEPES pH 7.4, 150 mM NaCl, 3 mM EDTA, 0.05% Tween-20, supplemented with 0.5 mg/mL BSA). The highest concentration of D614G-HexaPro and BA.1 spike was 32nM. Spike dilutions were then injected onto the chip surface from the lowest to the highest concentration without regeneration, including several buffer injections before the lowest non-zero concentration for signal stabilization. For each concentration, the data collection time for baseline, association and dissociation steps was 120 seconds, 300 seconds and 900 seconds, respectively. After the titration of each analyte, the chip surface was regenerated with two pulses (17 seconds per pulse) of 10mM Glycine, pH 2.0. IgGs and Fabs were re-captured at the start of each analyte cycle. The running buffer for all kinetic steps was 1xHBSTE-BSA. The titration data collected were processed with the Kinetics software package (Carterra), including reference subtraction, buffer subtraction and data smoothing. spike binding time courses for each antibody were fitted to a 1:1 Langmuir model to derive k_a, k_d and K_D values.

QUANTIFICATION AND STATISTICAL ANALYSIS

Statistical details, including numbers of replicates and measures of precision (standard error (SEM), can be found in the experimental methods, figure legends, figures, results, and methods.

Cell Reports, Volume *42*

Supplemental information

Bivalent intra-spike binding provides durability against emergent Omicron lineages: Results from a global consortium

Heather M. Callaway, Kathryn M. Hastie, Sharon L. Schendel, Haoyang Li, Xiaoying Yu, Jeremy Shek, Tierra Buck, Sean Hui, Dan Bedinger, Camille Troup, S. Moses Dennison, Kan Li, Michael D. Alpert, Charles C. Bailey, Sharon Benzeno, Jody L. Bonnevier, Jin-Qiu Chen, Charm Chen, Hyeseon Cho, Peter D. Crompton, Vincent Dussupt, Kevin C. Entzminger, Yassine Ezzyat, Jonathan K. Fleming, Nick Geukens, Amy E. Gilbert, Yongjun Guan, Xiaojian Han, Christopher J. Harvey, Julia M. Hatler, Bryan Howie, Chao Hu, Ailong Huang, Maya Imbrechts, Aishun Jin, Nik Kamachi, Gladys Keitany, Mark Klinger, Jay K. Kolls, Shelly J. Krebs, Tingting Li, Feiyan Luo, Toshiaki Maruyama, Michael A. Meehl, Letzibeth Mendez-Rivera, Andrea Musa, C.J. Okumura, Benjamin E.R. Rubin, Aaron K. Sato, Meiying Shen, Anirudh Singh, Shuyi Song, Joshua Tan, Jeffrey M. Trimarchi, Dhruvkumar P. Upadhyay, Yingming Wang, Lei Yu, Tom Z. Yuan, Erik Yusko, Bjoern Peters, Georgia Tomaras, and Erica Ollmann Saphire

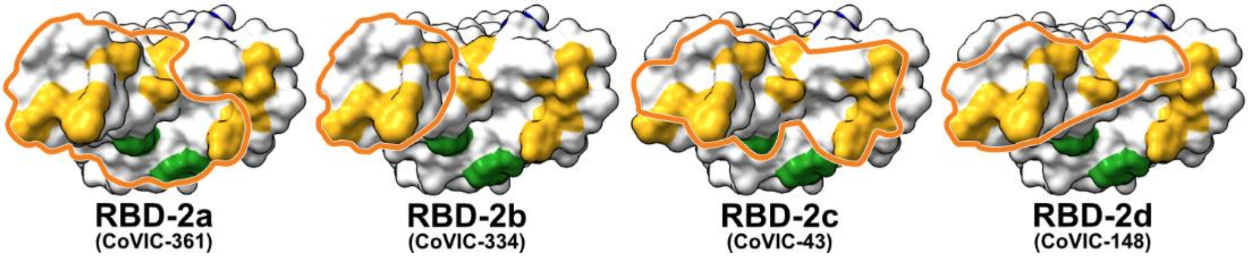

Figure S1. Antigenic sites of RBD-2 subgroups, related to Figure 1. Antigenic site footprints for RBD-2 subgroups RBD-2a, -2b, -2c, and -2d, approximated by docking a Fab into a negative stain electron microscopy map of spike bound to a representative antibody from the indicated epitope community, are highlighted by an orange outline.

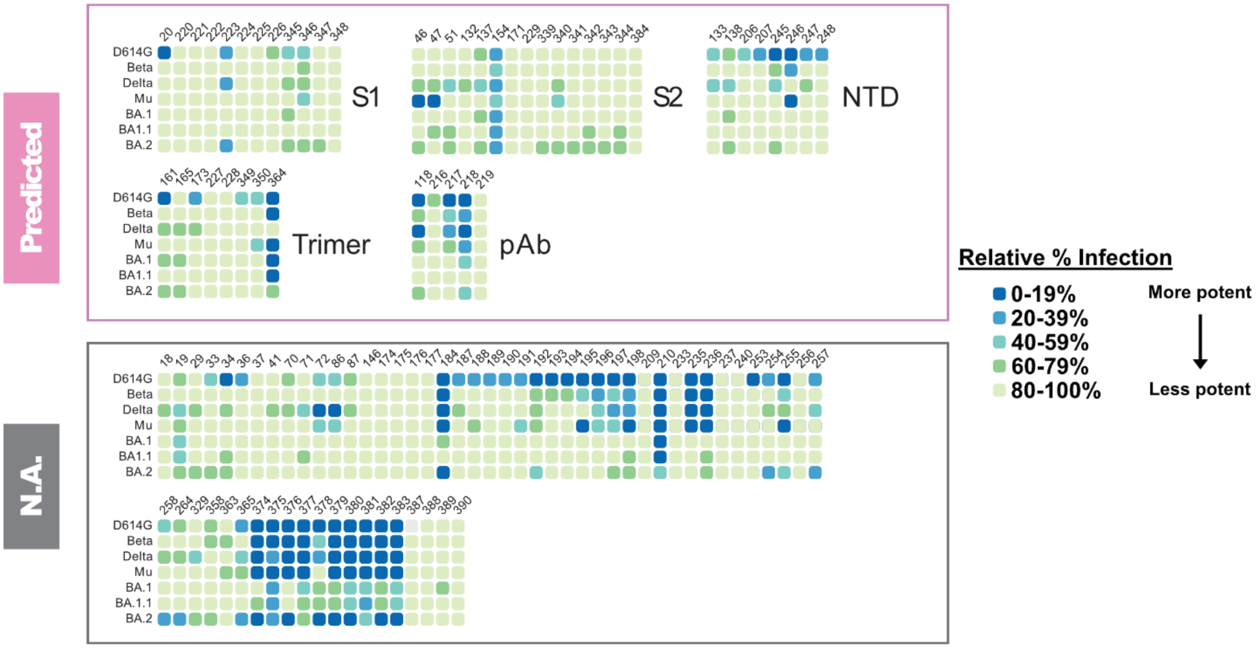

Figure S2. Block neutralization of SARS-CoV-2 pseudovirus bearing VoC spike proteins with additional CoVIC panel antibodies, related to Figure 2. Neutralization of SARS-CoV-2 pseudovirus with 250ng/mL antibody. Antibodies are organized by their predicted binding site or are uncategorized. Neutralization activity is expressed as the percentage of infected cells in the presence of antibody relative to cells infected in the absence of antibody.

A

RBD		IC50 (ng/mL) D614G	BA2.12.1	BA.4/5
1	COVIC-89	316.1	121.8	100.2
1	COVIC-93	26.6	30.8	39.0
1	COVIC-234	131.4	62.8	55.6
2a	COVIC-294	8.3	16.8	2.3
2a	COVIC-299	47.8	31.7	21.8
2a	COVIC-359	7.5	31.2	>>2,000
2a	COVIC-360	13.0	36.3	>>2,000
2a	COVIC-361	28.7	32.0	>2,000*
2a	COVIC-362	6.6	9.9	>2,000*
2b	COVIC-102	4.8	15.3	>2,000*
2b	COVIC-103	3.0	10.4	>2,000*
2b	COVIC-290	4.6	10.6	>2,000*
2b	COVIC-292	1.8	38.0	>>2,000
2b	COVIC-333	4.7	9.6	>>2,000
2b	COVIC-334	7.1	34.4	>2,000*
2b	COVIC-354	16.6	104.0	>>2,000
3a	COVIC-368	2.5	10.8	5.4
4a	COVIC-371	1.0	11.2	>2,000*
4b	COVIC-295	25.2	44.3	>2,000*
5d	COVIC-355	0.9		11.5

*Some neutralization observed at higher antibody concentrations

B

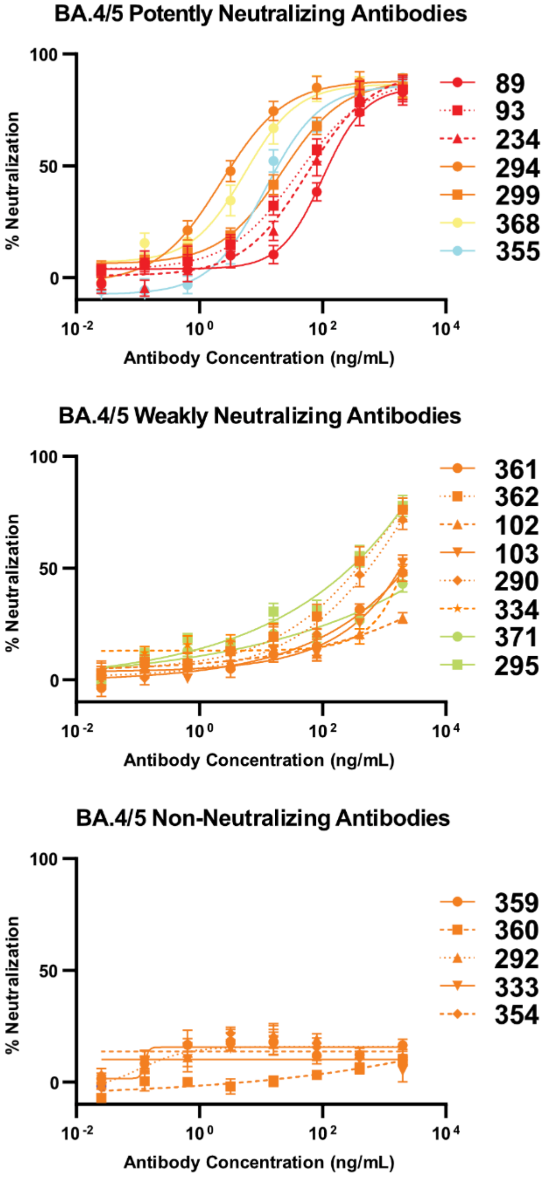

Figure S3. Omicron BA.4/5 and BA2.12.1 pseudovirus neutralization by select antibodies that potently neutralize Omicron BA.1, BA1.1, and BA.2, related to Figures 2 and 4. A) Neutralization titers against pseudovirus bearing D614G, BA2.12.1, and BA.4/5 spike for CoVIC antibodies that potently neutralized Omicron BA.1, BA1.1, and BA.2 in the block neutralization assay. **B)** Neutralization curves for BA.4/5 pseudoviruses. Error bars are ± SEM.

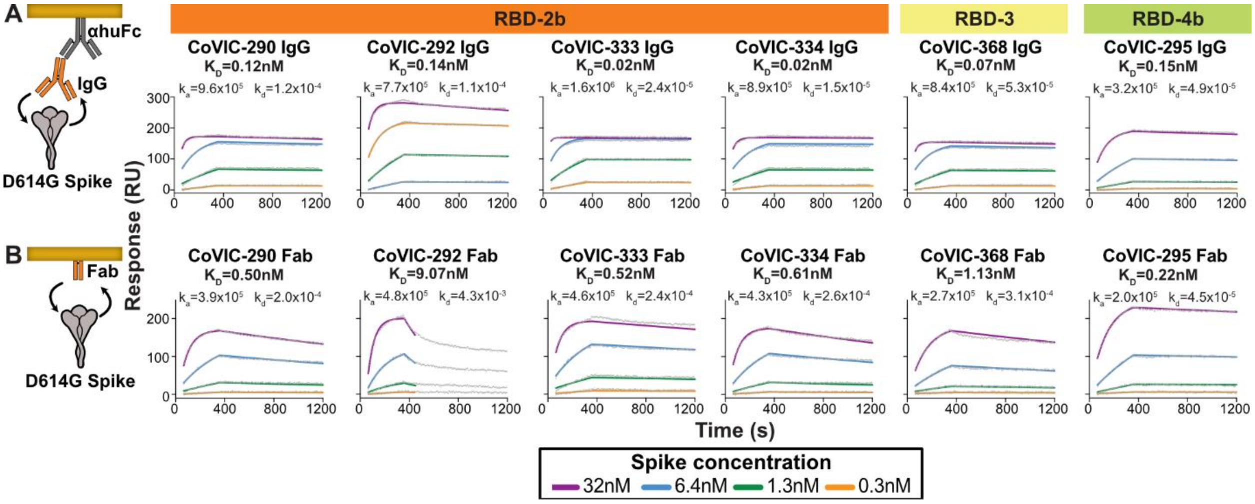

Figure S4. Binding kinetics of D614G spike ectodomains to IgG and Fabs, related to Figure 5. Surface plasmon resonance experiments comparing the binding kinetics of IgG **(A)** and Fab **(B)** to D614G spike. For each sample, experimental data (gray line) and 1:1 fitted curve (colored lines) are shown. Spike concentrations range from 0.3-32nM. K_D, k_a, and k_d values for each interaction are indicated.

Table S1. Neutralization activity of all items in the CoVIC panel against pseudovirus bearing major SARS-CoV-2 VoCs, related to Figures 2 and S2. Neutralization activity is expressed as the percentage of infected cells in the presence of 250ng/mL or 25μg/mL antibody relative to cells infected in the absence of antibody.
